# A personalized prediction model for distinguishing between asymptomatic bacteriuria and symptomatic urinary tract infections in patients with type 2 diabetes mellitus using machine learning

**DOI:** 10.3389/fendo.2025.1593735

**Published:** 2025-08-05

**Authors:** Shuangqing Liu, Juan Li, Yang Fang, Xiujuan Wu, Yang Cao, Keke Cai, Jing Yu, Yan Zhao, Yitao Duan

**Affiliations:** ^1^ Department of Clinical Laboratory, The Second Hospital of Tianjin Medical University, Tianjin, China; ^2^ Department of Respiratory, Characteristic Medical Center Of Chinese People’s Armed Police Force, Tianjin, China; ^3^ Department of Laboratory Medicine, the Third Affiliated Hospital of Zhengzhou University, Zhengzhou, China; ^4^ Zhengzhou Key Laboratory for In Vitro Diagnosis of Hypertensive Disorders of Pregnancy, Department of Laboratory Medicine, the Third Affiliated Hospital of Zhengzhou University, Zhengzhou, China; ^5^ People’s Hospital of Zhengzhou University, Heart Center of Henan Provincial People’s Hospital, Central China Fuwai Hospital, Central China Fuwai Hospital of Zhengzhou University, Zhengzhou, China; ^6^ Department of Urology, Tianjin Medical University Nankai Hospital, Tianjin, China; ^7^ Department of Urology, Tianjin Nankai Hospital, Tianjin, China; ^8^ Department of Endocrinology, the Second Hospital of Tianjin Medical University, Tianjin, China

**Keywords:** asymptomatic bacteriuria, type 2 diabetes mellitus, urinary tract infections, uropathogenic *Escherichia coli*, machine learning

## Abstract

**Background:**

Patients with type 2 diabetes mellitus (T2DM) have an increased susceptibility to urinary tract infections (UTIs), caused by uropathogenic *Escherichia coli* (UPEC). Asymptomatic bacteriuria (ASB) is a significant contributor, but lots of patients are difficult to distinguish. Distinguishing between ASB and symptomatic UTIs can greatly assist clinicians in rational use of antimicrobials.

**Methods:**

Patients with T2DM and UTIs caused exclusively by UPEC were recruited from the Second Hospital of Tianjin Medical University between 2018 and 2023. Demographic and clinical data were systematically collected for these patients through a retrospective electronic chart review, in accordance with the inclusion and exclusion criteria. We utilized this dataset as training set to develop an ASB predictive model called ASBPredictor.

**Results:**

A total of 337 cases were collected, comprising 158 cases (46.9%) of ASB and 179 cases (53.1%) of symptomatic UTIs. Based on the optimal predictive model, ASBPredictor exhibited a remarkable level of precision, achieving an area under the curve score of 0.82. The identification of ASB is influenced by several crucial factors, including urinary bacteria, urinary white blood cell clusters, C-reactive protein, alanine aminotransferase, glucose, gamma-glutamyl transpeptidase, sodium ions (Na^+^), and eosinophils.

**Conclusion:**

The ASBPredictor is an accurate, efficient, and reliable tool that helps doctors differentiate between ASB and symptomatic UTIs. This precise differential diagnosis has the potential to enhance the quality of antimicrobial prescribing.

## Introduction

Type 2 diabetes mellitus (T2DM) is a significant global health issue. According to a survey conducted by the International Diabetes Federation (IDF) in 2021, there are approximately 537 million DM patients worldwide, and this number is predicted to rise to 783 million by 2045 (1). T2DM accounts for the vast majority (> 90%) of diabetes worldwide (2, 3). Urinary tract infections (UTIs) are the second most common infection among hospitalized patients (4, 5), which are commonly caused by uropathogenic *Escherichia coli* (UPEC) (6–8). UTIs in patients with T2DM are more than 4 times as common as in normal people (9), and the increased risk of UTIs in individuals with T2DM can be attributed to various factors, including hyperglycemia, impaired immune function, and structural changes in the urinary tract (10).

The term ‘UTIs’ typically includes both symptomatic UTIs and asymptomatic bacteriuria (ASB). In patients with T2DM, ASB typically has a higher prevalence compared to symptomatic UTIs (11). Multiple guidelines (12, 13) typically recommend against intervening in cases of ASB, except for pregnant individuals (14) and patients requiring urological surgery, compared to symptomatic UTIs. Instead, treating ASB may expose patients to the risks associated with antimicrobials, including adverse drug reactions and antimicrobials resistance, potentially leading to prolonged hospital stays for those who are hospitalized (15–17). Despite the existence of guidelines (12, 13) and measures (15, 18–20) aimed at improving the management of ASB, up to 80% hospitalized patients with ASB are still treated with antimicrobials (4). In addition to doctors having limited clinical experience (21) and not following guidelines, another significant factors is the difficulty in distinguishing between UTIs and ASB based on clinical symptoms in some patients, such as those with vague consciousness or unclear expression (22, 23), elderly patients with decreased sensitivity (24, 25), and patients with prostatitis (26) or prostatic hyperplasia (27). Several biomarkers have been researched to assist clinicians in identifying ASB, but their effectiveness is limited (28, 29).

Machine learning (ML) algorithms can identify patterns and risk factors, leading to earlier and more accurate diagnoses and personalized treatment plans. In case of UTIs, ML has been used to predict UTI presence (30) and antimicrobial susceptibility test (AST) results (31). For instance, Xiong et al. achieved a remarkable area under the curve (AUC) score of 0.979 in predicting UTIs in T2DM by employing a gradient boosting algorithm (32). Nevruz et al. found that a random forest algorithm had the highest accuracy in predicting uropathogen antimicrobial resistance, with AUCs ranging from 0.777 to 0.884 for different antimicrobials (33). Other models like XGBoost (34), PittUDT (35), and NoMicro models (36) have also been trained. However, there is currently no research on ML for differentiating ASB from symptomatic UTIs. The objective of this study is to develop a personalized ASBPredictor model that can accurately distinguish between ASB and symptomatic UTIs by using a comprehensive clinical variables dataset. The implementation of this model has the potential to improve clinical decision-making, reduce unnecessary antimicrobial usage, and lower healthcare costs.

## Materials and methods

### Data collection and preprocessing

In this study, T2DM UTI patients infected only by UPEC were recruited from the Second Hospital of Tianjin Medical University (Tianjin, China) between 2018 and 2023. Patients with positive urine cultures for UPEC were diagnosed with either symptomatic UTIs or ASB, depending on whether they had signs or symptoms meeting UTI diagnostic criteria (12, 13). Specifically, ASB patients could not have any of the following documented signs or symptoms: dysuria, urinary frequency/urgency, suprapubic pain, fever (temperature ≥ 38°C), costovertebral pain/tenderness, hematuria, autonomic dysreflexia, or increased spasticity in patients with spinal cord injury. Patients with acute alterations in mental status often cannot communicate symptoms and were categorized as suffering ASB if they had none of the aforementioned signs or symptoms and no systemic signs of possible infection. Otherwise, the patients would be assigned to the symptomatic UTIs group. Meanwhile, patients were not eligible for inclusion if they met any of the following criteria: (1) pregnant; (2) urinary stent, nephrostomy, altered urinary tract anatomy, or urologic surgery before UC; (3) intensive care unit (ICU) admission within 3 days before or after UC; (4) concomitant infection that results in unclear UTIs symptoms; (5) active treatment and/or prophylaxis for UTIs on admission (4).

Demographic and clinical data were systematically collected for the two groups of patients by retrospective electronic chart review. The following clinical data were recorded: (1) the diabetes control condition and complications; (2) the presence of typical urinary tract symptoms (dysuria, increased urinary frequency, urgency, etc.); (3) all laboratory results obtained on the day (± 1 day) of the first urine sample testing positive for UPEC; (4) antimicrobial sensitivity results for the first urine culture-positive UPEC isolate, which were interpreted using the breakpoints outlined in the 2023 Clinical & Laboratory Standards Institute guidelines.

The data were preprocessed to ensure accuracy and consistency. First, missing values were identified and imputed using the mean values separately calculated for the ASB and symptomatic UTI groups. Next, outliers were detected and removed using statistical methods. Finally, the data were standardized to eliminate the influence of measurement units and enhance comparability between variables. To address missing values in the dataset, we evaluated two imputation strategies: mean imputation and K-nearest neighbors (KNN) imputation. Mean imputation involved replacing missing values with the mean values calculated separately for the ASB and symptomatic UTI groups. KNN imputation utilized the k-nearest neighbors algorithm to estimate missing values based on similar patients’ data patterns. A comparative analysis was performed to determine the optimal imputation method for our dataset (**Supplementary Figure 1**). Mean imputation demonstrated superior performance with an area under the ROC curve of 0.82 and precision-recall AUC of 0.78, compared to KNN imputation which achieved ROC AUC of 0.70 and PR AUC of 0.65. Based on these results, mean imputation was selected as the primary imputation strategy for the ASBPredictor model development. The processed dataset used for machine learning training is provided in **Supplementary Table 1**.

To ensure data quality, differential diagnosis of ASB, data entry, and cleaning were performed by two independent researchers. In cases of discrepancies, a third researcher was consulted to resolve differences. All data were stored securely and analyzed using appropriate statistical software.

### External validation dataset

To assess the temporal generalizability of the ASBPredictor model, we collected an independent validation dataset from the same institution covering the period from January 1, 2024, to June 25, 2025. This validation cohort included 103 patients, applying identical inclusion and exclusion criteria as the training dataset. The same data collection procedures, laboratory measurement protocols, and clinical assessment methods were employed to ensure consistency. Detailed characteristics of the validation dataset are provided in **Supplementary Table 2**.

### Feature selection

Feature selection was meticulously carried out to identify the most significant predictors of ASB versus symptomatic UTIs. We employed a variety of methods for this purpose, including correlation analysis, univariate logistic regression, and recursive feature elimination (RFE). Antibacterial drug sensitivity and laboratory features demonstrating strong correlations with the outcome variable were preserved, whereas those exhibiting minimal correlations were excluded from further analysis. In addition to these techniques, advanced methods such as Shapley Additive exPlanations (SHAP) (37) and Uniform Manifold Approximation and Projection for Dimension Reduction (UMAP) (38) were utilized to further elucidate the relationships and importance of the selected features.

### Machine learning methods

In this investigation, we utilized an array of machine learning algorithms to accurately predict the diagnosis based on the extracted features. The algorithms tested included Support Vector Machine (SVM), Decision Trees, Random Forest, K-Nearest Neighbors (KNN), Neural Networks, XGBoost, LightGBM. These models were implemented using the SciKit-learn library in Python (39).

We systematically evaluated seven state-of-the-art machine learning algorithms to identify the optimal approach for ASB prediction (**Table 1**). The algorithm selection encompassed traditional machine learning methods (SVM, Decision Trees, KNN), ensemble methods (Random Forest), deep learning approaches (Neural Networks), and advanced gradient boosting frameworks (XGBoost, LightGBM).

**Table 1:** Machine learning algorithms evaluated in ASBPredictor development.

Algorithm	Principle	Advantages	Disadvantages	Performance in study
Support Vector Machine (SVM)	Finds optimal hyperplane to separate classes using kernel trick	1. Effective in high dimensions 2. Memory efficient 3.Versatile kernel functions 4.Good generalization	1. Poor performance on 2. large datasets 3. Sensitive to feature scaling 4. No probabilistic output 5.Difficult to interpret	ROC AUC: 0.62 PR AUC: 0.58
Decision Trees	Creates tree-like model of decisions based on feature values	1.Highly interpretable 2.Handles both numerical 3.and categorical data 4.No assumptions about 5.data distribution 6.Feature selection built-in	1.Prone to overfitting Unstable (high variance) 2.Biased toward features with many levels 3.Poor performance on linear relationships	ROC AUC: 0.57 PR AUC: 0.66
Random Forest	Ensemble of decision trees with bootstrap aggregating and random feature selection	1.Reduces overfitting 2.Handles missing values 3.Provides feature importance 4.Good performance without tuning 5.Handles large datasets well	1.Less interpretable than single trees 2.Memory intensive 3.May overfit with very noisy data 4.Biased toward 5.categorical variables with many categories	ROC AUC: 0.82 PR AUC: 0.78 Selected as optimal model
K-Nearest Neighbors (KNN)	Classifies based on majority class of k nearest neighbors in feature space	1.Simple and intuitive 2.No assumptions about data distribution 3.Effective for local patterns 4.Naturally handles multi-class problems	1.Computationally expensive for large datasets 2.Sensitive to irrelevant features 3.Requires feature scaling 4.Poor performance in high dimensions	ROC AUC: 0.67 PR AUC: 0.67
Neural Networks	Multi-layer perceptrons with non-linear activation functions	1.Captures complex non-linear relationships Universal function 2.approximator 3.Flexible architecture Good performance on large datasets	1.Black box (low interpretability) 2.Requires large datasets 3.Prone to overfitting Sensitive to 4.hyperparameters Computationally intensive	ROC AUC: 0.70 PR AUC: 0.61
XGBoost	Gradient boosting framework with advanced regularization and optimization	1.High predictive performance 2.Handles missing values 3.Built-in regularization 4.Feature importance scores 5.Efficient implementation	1.Many hyperparameters to tune 2.Can overfit with small datasets 3.Less interpretable than simple models 4.Sensitive to outliers	ROC AUC: 0.79 PR AUC: 0.75
LightGBM	Gradient boosting framework optimized for speed and memory efficiency	1.Fast training speed 2.Lower memory usage 3.High accuracy 4.Handles categorical features natively 5.Good parallel learning support	1.Prone to overfitting on small datasets 2.Sensitive to hyperparameters 2.May be unstable with small datasets 4.Less mature than XGBoost	ROC AUC: 0.77 PR AUC: 0.73

Each algorithm was chosen based on its specific strengths and applicability to clinical data. Traditional methods provided interpretability and baseline performance, ensemble methods offered improved robustness and reduced overfitting, while gradient boosting frameworks provided state-of-the-art predictive performance. The comprehensive evaluation ensured that the selected model (Random Forest, ROC AUC = 0.82) represented the optimal balance between predictive accuracy, interpretability, and clinical applicability for the ASBPredictor system.

Each model was meticulously trained utilizing a subset of selected features enhanced through hyperparameter optimization. Their performance was assessed using a suite of evaluation metrics, including accuracy, precision, recall, F1 score, and the AUC. By analyzing the performance outcomes across these diverse algorithms, we were able to ascertain the most efficacious model for discriminating ASB from symptomatic UTIs. To address potential class imbalance concerns and ensure model robustness, we evaluated the Synthetic Minority Oversampling Technique (SMOTE) for data augmentation. Although our dataset showed relatively balanced distribution between ASB (46.9%) and symptomatic UTI (53.1%) cases, we performed comparative analysis using SMOTE to generate synthetic samples and assess potential performance improvements. The SMOTE analysis (**Supplementary Figure 2**) demonstrated nearly identical performance between the original Random Forest model (ROC AUC = 0.86, PR AUC = 0.83) and the SMOTE-augmented model (ROC AUC = 0.86, PR AUC = 0.85), indicating that our original model was not significantly affected by class imbalance.

### Evaluation metrics

In our research, the primary objective was to predict the likelihood of either ASB or symptomatic UTIs using a robust machine learning framework. We implemented a tenfold cross-validation technique to ensure the model’s reliability and generalizability. The effectiveness of our predictive model was quantitatively measured using several key evaluation metrics: the true positive rate (TPR, recall) (1), false positive rate (FPR) (2), and positive predictive value (PPV, precision) (3) calculate as follows:


(1) $$Recall\;=\;TPR\;=\;\frac{TP}{TP+FN}\;$$

(2) $$FPR\;=\;\frac{FP}{FP+TN}$$

(3) $$Precision\;=\;PPV\;=\;\frac{TP}{TP+FP}$$

## Results

### Overview of ASBPredictor performance and selection criteria

**Figure 1:**
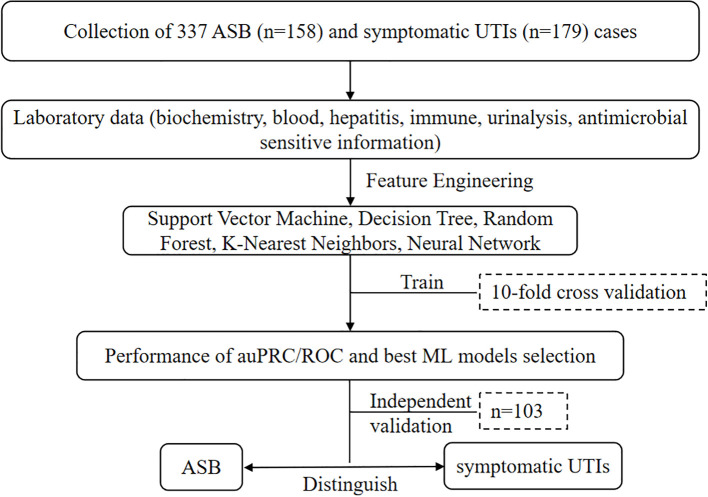
Performance metrics and machine learning model selection flowchart for distinguishing ASB and symptomatic UTIs using various classification techniques and comprehensive laboratory datasets.

In the study, a total of 337 cases were collected, comprising 158 cases (46.9%) of ASB and 179 (53.1%) cases of symptomatic UTIs. Extensive feature engineering was conducted utilizing laboratory data, and the ML models mentioned above were evaluated. These models underwent a rigorous 10-fold cross-validation process to ascertain their performance. The evaluation metrics focused on the area under the precision-recall curve (auPRC) and receiver operating characteristic (ROC) curve to select the best performing model. The selected model demonstrated promising capabilities in distinguishing between ASB and symptomatic UTIs effectively (**Figure 1**).

### Clinical and laboratory features

**Figure 2:**
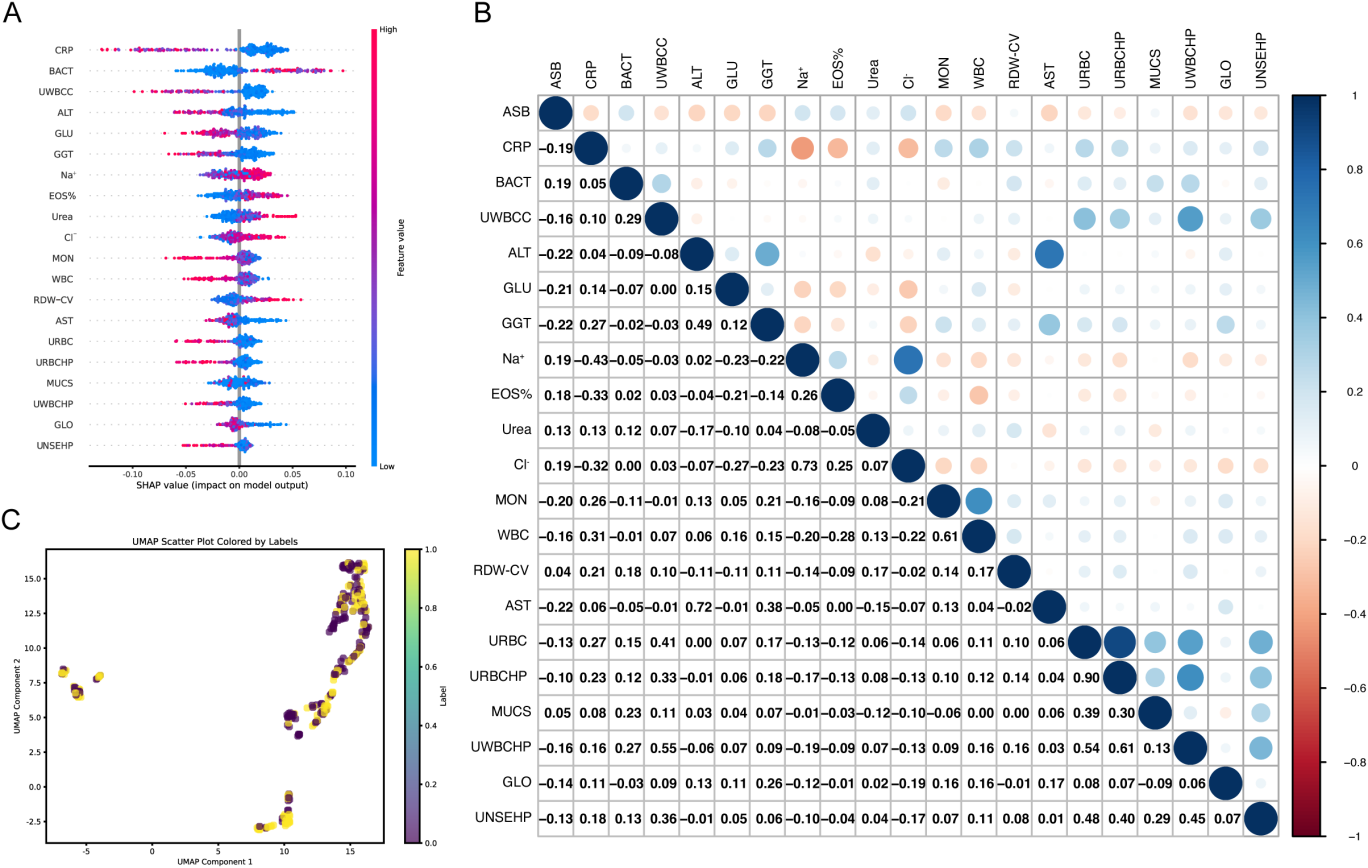
Visualization of Machine Learning Analysis Predicting the Presence of ASB. **(A)** SHAP summary plot demonstrating the impact of individual features on the prediction model. The color gradient from blue to red indicates the value magnitude of each feature. **(B)** Correlation heatmap showing Pearson correlation coefficients between SHAP values of features. The color scale transitions from red (negative correlation) to blue (positive correlation), illustrating both synergistic and antagonistic relationships among features influencing model predictions. **(C)** UMAP scatter plot showcasing the clustering of patient data based on drug sensitivity and laboratory markers.

The Shapley values (SHAP) and UMAP were utilized to explain the feature selected. The SHAP summary plot provides insights into the contribution of each feature towards the prediction of ASB presence. Features such as C-reactive protein (CRP), bacterial particle of automated urine flow cytometry (BACT), urine white blood cell clusters (UWBCC), alanine aminotransferase (ALT) and blood glucose (GLU) show higher SHAP values, suggesting a significant impact on the model’s output (**Figure 2A**). **Figure 2B** illustrates the correlation between various features and ASB based on SHAP values, offering deeper insights into the relationships between the variables and ASB. Upon analysis, certain features, such as BACT, sodium ions (Na^+^), and eosinophil percentage (EOS%), displayed a strong positive correlation with ASB, while negative correlations were observed for CRP and GLU. These findings suggest that these variables may collectively contribute to the predictive capabilities of the model ASBPredictor. This UMAP scatter plot visualizes the multidimensional data used to predict the presence ASB of developing (**Figure 2C**). The plot reveals distinct clusters, indicating potential subgroups among patients based on their laboratory profiles.

### Performance of machine learning models

**Figure 3:**
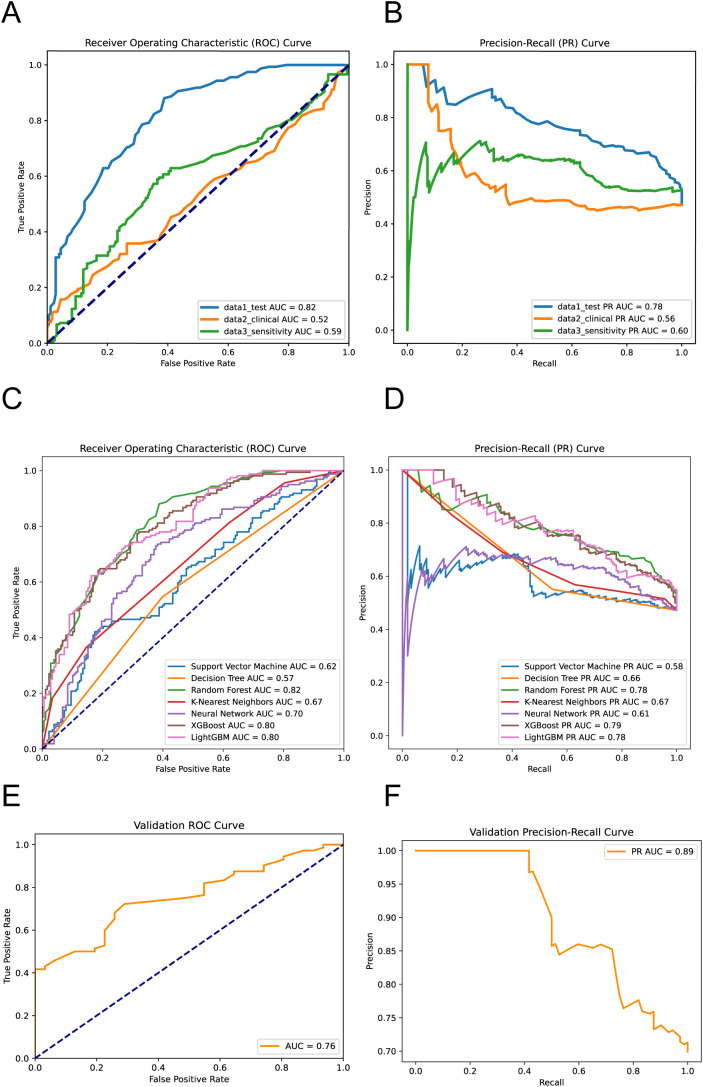
Performance evaluation of the machine learning models. ROC Curves (A) and PR Curves (B) for virous dataset performance. ROC Curves (C) and PR Curves (D) for different machine learning models predicting ASB. External validation results showing ROC Curve (E) and PR Curve (F) on the independent temporal validation dataset (n=103, January 2024 - June 2025) (Equations 1–3).

The AUC values from the ROC analysis are 0.82, 0.52, and 0.59 for data1_test (laboratory dataset), data2_clinical (clinical dataset), and data3_sensitivity (antimicrobial sensitivity dataset), respectively (**Figure 3A**). These values demonstrate that the laboratory dataset exhibits the highest capability in cases evaluation, as evidenced by its superior true positive rate against an increasing false positive rate. For the precision-recall curves (PR curves), the AUC scores are 0.78 for data1_test, 0.56 for data2_clinical, and 0.60 for data3_sensitivity (**Figure 3B**). These results highlight that the ASBPredictor using data1 not only predicts more true positives but also maintains a commendable precision across the predicted positives, which is vital in clinical applications where avoiding false negatives is critical. Interestingly, the joint detection did not show significant superiority. This portion underscores the efficacy of integrating specific sensitivity features into the predictive models, enhancing their diagnostic precision for identifying patients likely to develop ASB.

The ROC analysis reveals the following AUC values: SVM at 0.62, Decision Tree at 0.57, Random Forest at 0.82 (**Supplementary Figure 3**), KNN at 0.67, and Neural Network at 0.70, XGBoost at 0.80 and LightGBM at 0.80. Notably, the Random Forest model demonstrates the highest capability in differentiating between ASB and symptomatic UTIs cases, indicative of its robustness in handling this predictive task (**Figure 3C**). The PR curve analysis further supports these findings with AUC values as follows: SVM at 0.58, Decision Tree at 0.66, Random Forest at 0.78, KNN at 0.67, Neural Network at 0.61, XGBoost at 0.79 and LightGBM at 0.78 (**Figure 3D**). These results suggest that the Random Forest model not only predicts a higher proportion of true positives but also maintains higher precision across its predictions, which is particularly crucial for clinical settings where the consequences of false negatives can be significant. This segment of the analysis highlights the superior performance of the Random Forest algorithm over others, confirming its effectiveness in leveraging complex patterns and interactions within the data to improve diagnostic accuracy for ASB.

To evaluate the temporal generalizability of ASBPredictor, we tested the trained model on an independent validation dataset of 103 patients collected from January 2024 to June 2025. The validation results demonstrated robust model performance with an ROC AUC of 0.76 and PR AUC of 0.89 (**Figures 3E, F**). These validation metrics, while slightly lower than the training performance (ROC AUC: 0.82, PR AUC: 0.78), remain within an acceptable range and suggest good temporal stability of the model’s predictive capabilities.

### Important features in ASB and symptomatic UTIs

**Figure 4:**
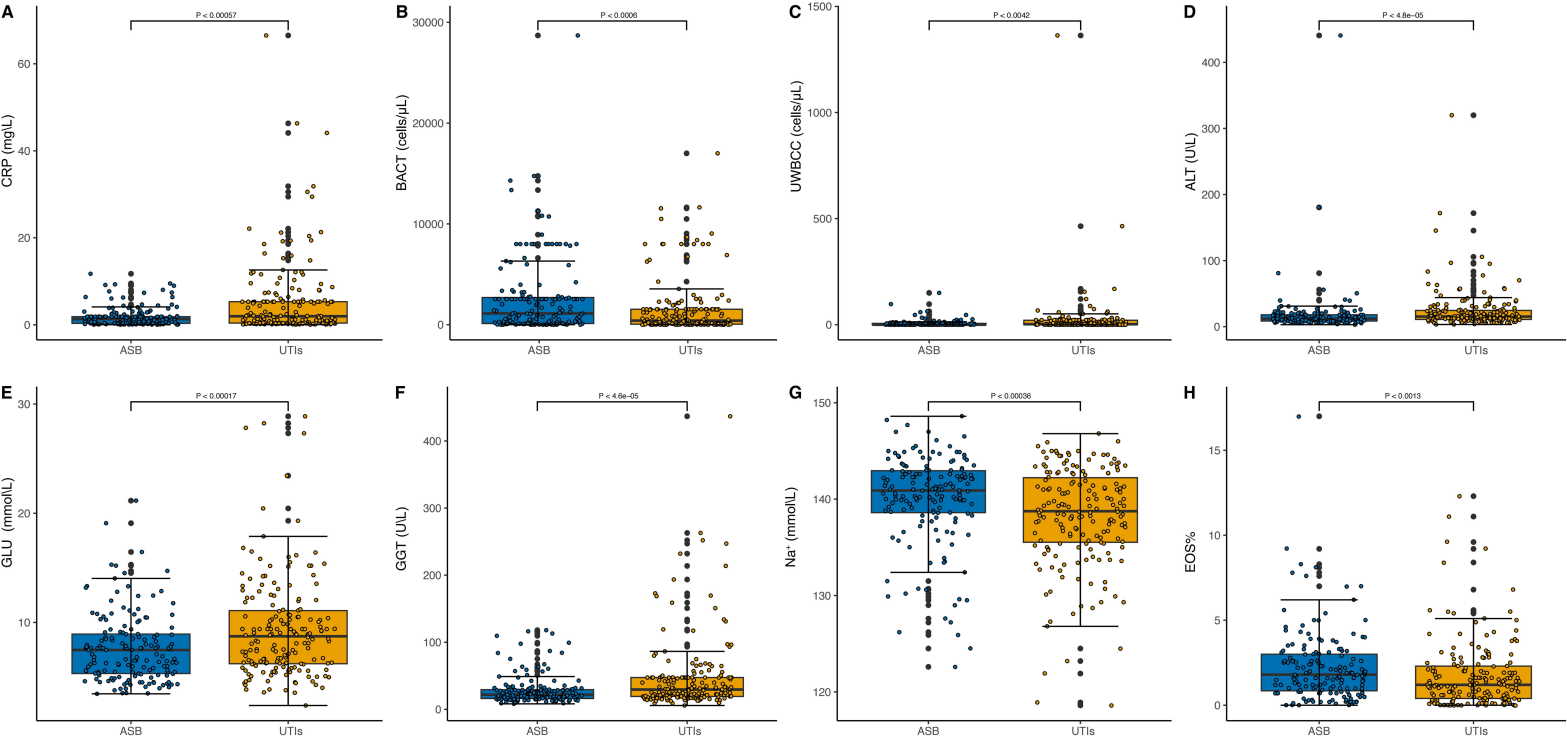
Distribution of Biomarker Concentrations in ASB and symptomatic UTI Cases. In panels labeled **(A–H)**, the Y-axis represents biomarker concentrations while the X-axis categorizes the conditions, distinguishing between ASB and symptomatic UTIs. The median concentrations of the biomarkers are annotated in each plot, highlighting the significant differences in biomarker levels between the two patient groups. CRP **(A)**, BACT **(B)**, UWBCC **(C)**, ALT **(D)**, GLU **(E)**, GGT **(F)**, Na^+^ **(G)** and EOS% **(H)**.

**Figure 4** presents the comparative distribution of biomarker concentrations between patients with ASB and those diagnosed with symptomatic UTIs. The Y-axis has been carefully scaled to effectively highlight the broad spectrum of biomarker concentrations, clearly delineating the distinctions between the ASB group and the symptomatic UTI group. Notably, BACT, Na^+^, and EOS% show significantly higher levels in ASB cases compared to symptomatic UTIs, whereas the remaining five biomarkers (CRP, UWBCC, ALT, GLU, and GGT) exhibit markedly elevated concentrations in symptomatic UTIs than in ASB. These findings highlight the substantial variability in biomarker levels between ASB and symptomatic UTIs, further reinforcing their diagnostic relevance.

### Clinical case examples demonstrating ASBPredictor decision-making

**Figure 5:**
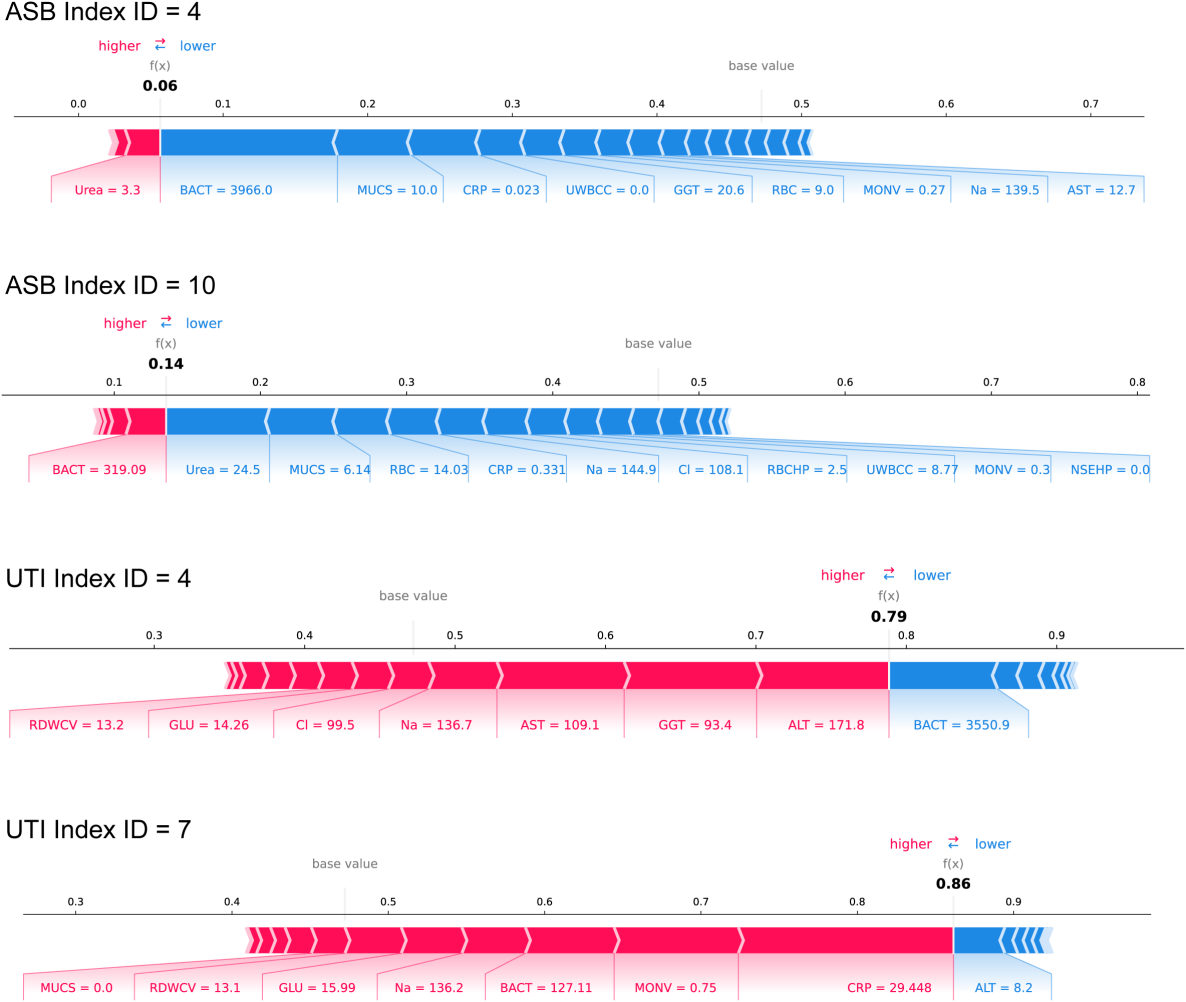
Clinical case examples demonstrating ASBPredictor decision-making process using SHAP waterfall plots. Four representative patient cases are shown: two ASB cases (Index ID 4 and 10) and two symptomatic UTI cases (Index ID 4 and 7).

To enhance clinical interpretability of the ASBPredictor model, **Figure 5** presents four representative patient cases with individual SHAP waterfall plots illustrating how specific laboratory parameters contribute to the differentiation between ASB and symptomatic UTIs. Two ASB cases (Index ID 4 and 10) demonstrate prediction scores of 0.06 and 0.14 respectively, characterized by high bacterial counts (BACT = 3966.0 and 319.09) combined with minimal inflammatory responses (low CRP and UWBCC values), supporting asymptomatic bacteriuria diagnosis. In contrast, two symptomatic UTI cases (Index ID 4 and 7) show prediction scores of 0.79 and 0.86, driven by elevated inflammatory markers including high CRP (29.448), poor glycemic control (GLU = 14.26-15.99), and liver enzyme elevation (ALT = 171.8-8.2, AST = 109.1), indicating active systemic infection. The SHAP waterfall plots provide clinicians with transparent, feature-by-feature explanations of model predictions, where red bars indicate factors favoring symptomatic UTI diagnosis and blue bars support ASB classification. This interpretable approach enables healthcare providers to understand the underlying clinical reasoning behind each prediction, validate model decisions against clinical judgment, and confidently apply the ASBPredictor in routine practice for optimizing antimicrobial stewardship decisions.

## Discussion

To the best of our knowledge, there is currently no utilization of machine learning algorithms for predicting ASB. The ASBPredictor utilizes a few laboratory indicators to accurately predict the likelihood of ASB by a simple and convenient script. It can assist doctors in making more accurate judgments about ASB, avoiding the uncertainty caused by symptom descriptions. With the intuitiveness and interpretability, ASBPredictor can monitor the changes of laboratory data to predict the progression of ASB, thereby prompting clinical doctors to take intervention measures timely. It can also increase the efficiency of hospital managers in ASB management.

The ASBPredictor model in T2DM incorporates several important features, including inflammatory indicators (CRP), urinary indicators (BACT and UWBCC), biochemical indicators [ALT, GLU, (gamma-glutamyl transpeptidase, GGT), Na^+^], and blood routine indicators (EOS%). In patients with T2DM, the majority of UTIs, including ASB, are characterized by increased levels of infection markers in urinalysis (40). This study further suggests that BACT could be a promising indicator for ASB, while UWBCC appears to be more closely associated with symptomatic UTIs (**Figures 4B, C**). Bacterial virulence genes show no significant differences between the two groups (data not shown); the bacterial load disparity between ASB and UTIs may instead stem from variations in human immune status, resulting in higher tolerance of colonization in ASB but greater sensitivity to active infection in UTIs. Additionally, patients with symptomatic UTIs tend to have elevated blood sugar levels compared to individuals with ASB (**Figure 4E**). Inadequate blood glucose control can lead to increased glucose levels in the urine, creating a more conducive environment for bacterial growth in the urinary tract, thereby raising the risk of UTIs (41, 42). Furthermore, symptomatic UTIs show a stronger correlation with low sodium levels and elevated CRP levels (**Figures 4A, E**). Consistent with previous findings, significant associations between CRP levels and hyponatremia (Na^+^ < 135 mmol/L) have been identified (43, 44), and CRP’s diagnostic utility for UTIs has been confirmed by multiple studies (45, 46). It could be understood that CRP (and WBC count as mentioned above), traditionally associated with inflammation, is particularly relevant to symptomatic UTIs which involve a robust host inflammatory response, unlike ASB. Moreover, ALT and GGT exhibit higher concentrations in symptomatic UTIs (**Figures 4D, F**). However, there are limited reports suggesting that UTIs themselves (47) as well as the use of antimicrobials such as nitrofurantoin (48), cephalosporins (49), quinolones (50), can lead to liver damage and elevate enzymes such as ALT and GGT. Further observation and validation are still needed to determine the impact of these two indicators on ASB and symptomatic UTIs. Lastly, patients with symptomatic UTIs generally have lower EOS% compared to individuals with ASB (**Figure 4H**). However, there is currently no research examining the correlation between EOS% and UTIs, which necessitates further investigation.

This study has several inherent limitations. First, the stringent data filtering process limits the generalizability of our model. Additionally, missing data for inflammatory factors may affect the accuracy of the predictions. Finally, the predictive model has not yet been externally validated, which is a crucial step in assessing its generalizability and reliability.

Overall, the ASBPredictor effectively predicts the likelihood of ASB using various clinical and laboratory indicators, leveraging machine learning algorithms. This approach has the potential to reduce unnecessary antimicrobial use and lower healthcare costs.

## Data Availability

The datasets presented in this study can be found in online repositories. The names of the repository/repositories and accession number(s) can be found in the article/**Supplementary Material**.

## Supplementary material

Supplementary Figure 1 Comparison of imputation methods for missing data handling in the ASBPredictor model. ROC curves (left panel) and Precision-Recall curves (right panel) comparing the performance of mean imputation versus K-nearest neighbors (KNN) imputation strategies (Equations 1–3).

Supplementary Figure 2 Performance comparison between original Random Forest model and SMOTE-augmented Random Forest model for ASB prediction (Equations 1–3).

Supplementary Figure 3 The Random Forest method 10-fold cross-validation methodology process (Equations 1, 2).

Supplementary Table 1 The trained data of laboratory features.

Supplementary Table 2 The Validation data of laboratory features.
