# Supplementary figures and images for: A personalized prediction model for distinguishing between asymptomatic bacteriuria and symptomatic urinary tract infections in patients with type 2 diabetes mellitus using machine learning
Source: Front Endocrinol (Lausanne). 2025 Aug 5;16:1593735. doi: 10.3389/fendo.2025.1593735 (PMC12361136; doi:10.3389/fendo.2025.1593735)

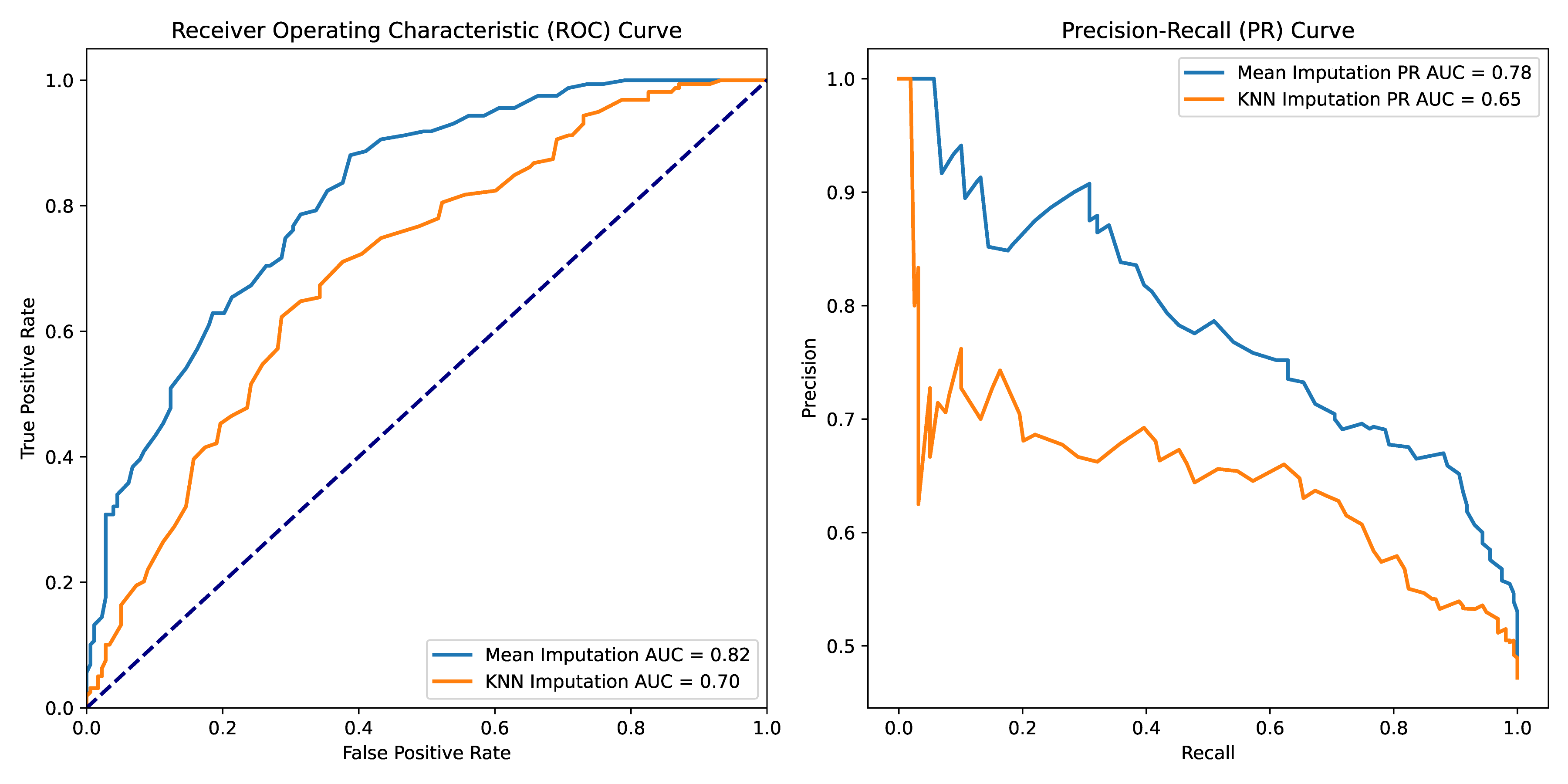

Supplement: Supplementary Figure 1 — Comparison of imputation methods for missing data handling in the ASBPredictor model. ROC curves (left panel) and Precision-Recall curves (right panel) comparing the performance of mean imputation versus K-nearest neighbors (KNN) imputation strategies (Equations 1–3). [file SupplementaryFile1.tif]

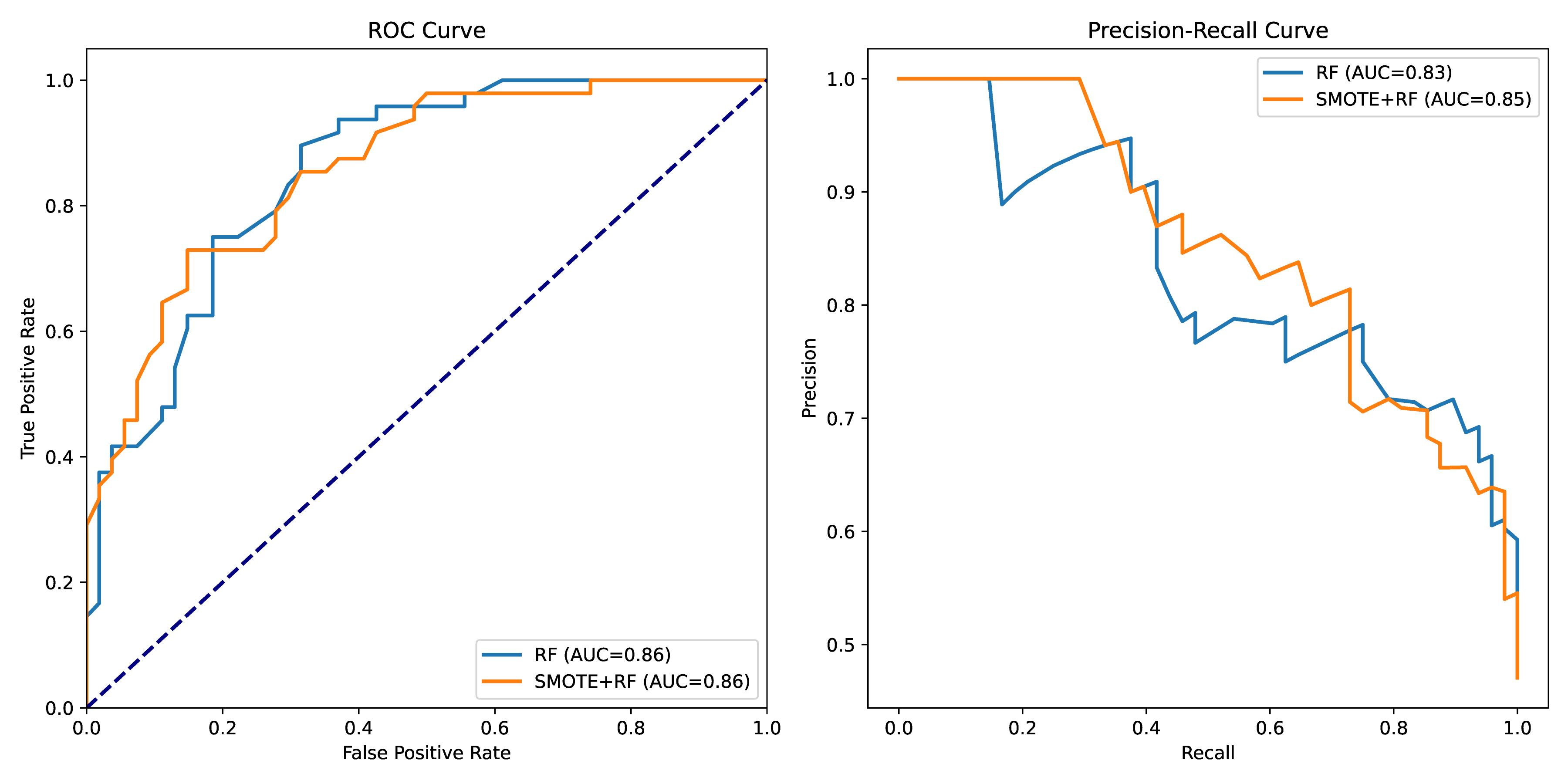

Supplement: Supplementary Figure 2 — Performance comparison between original Random Forest model and SMOTE-augmented Random Forest model for ASB prediction (Equations 1–3). [file SupplementaryFile2.tif]

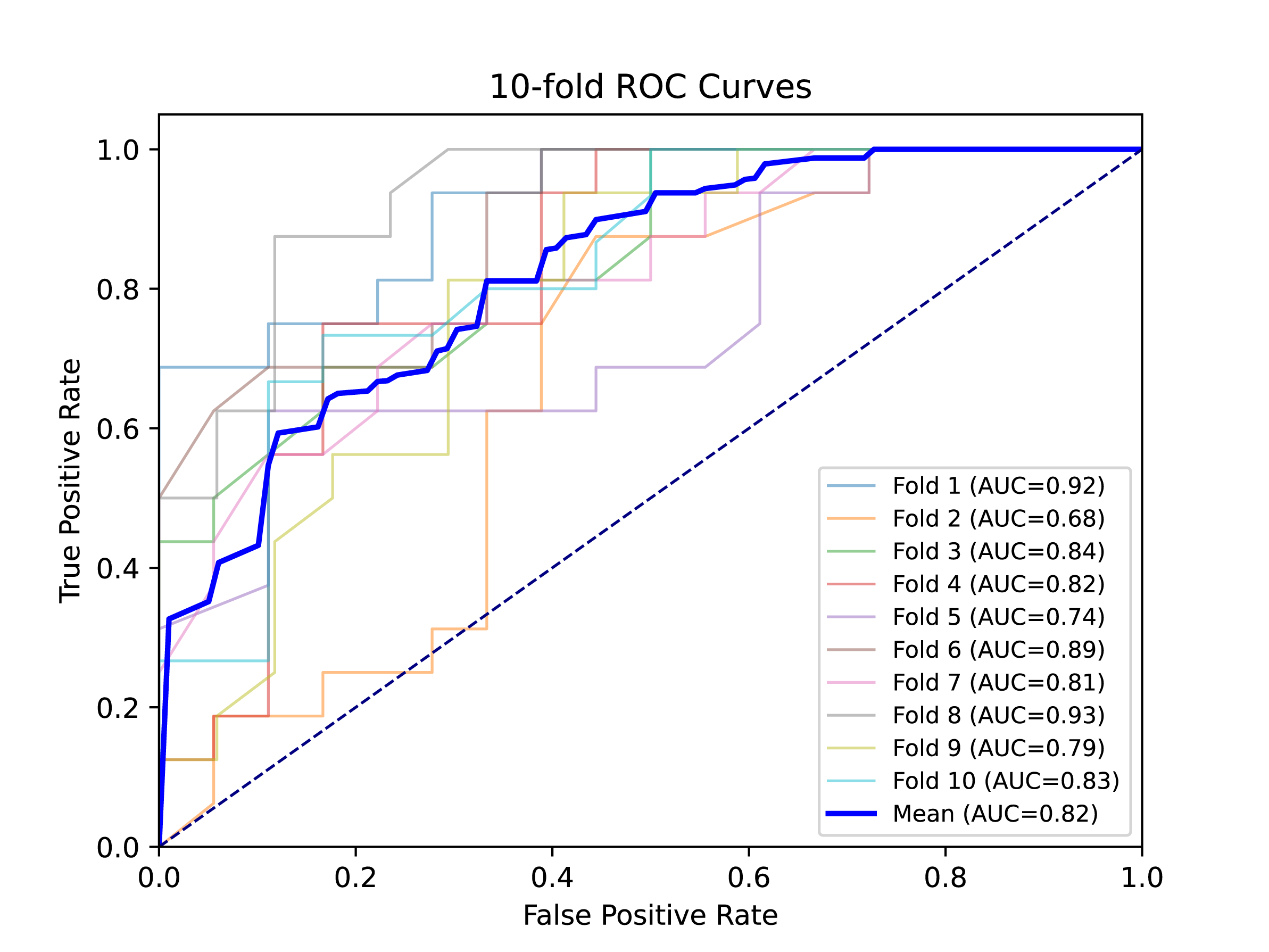

Supplement: Supplementary Figure 3 — The Random Forest method 10-fold cross-validation methodology process (Equations 1, 2). [file SupplementaryFile3.tif]
