# Supplementary material for: A personalized prediction model for distinguishing between asymptomatic bacteriuria and symptomatic urinary tract infections in patients with type 2 diabetes mellitus using machine learning
Source: Front Endocrinol (Lausanne). 2025 Aug 5;16:1593735. doi: 10.3389/fendo.2025.1593735 (PMC12361136; doi:10.3389/fendo.2025.1593735)
Supplement: Supplementary Table 1 — The trained data of laboratory features. [file Table1.docx]

| label | CRP | BACT | UWBCC | ALT | GLU | GGT | Na+ | EOS% | Urea | Cl- | MON | WBC | RDW-CV | AST | URBC | URBCHP | MUCS | UWBCHP | GLO | UNSEHP |
| --- | --- | --- | --- | --- | --- | --- | --- | --- | --- | --- | --- | --- | --- | --- | --- | --- | --- | --- | --- | --- |
| 1 | 1.87 | 25.47 | 0.88 | 18.39 | 14.72 | 28.76 | 141.60 | 1.20 | 5.80 | 109.70 | 0.30 | 4.80 | 11.90 | 25.32 | 135.23 | 24.07 | 0.00 | 90.03 | 26.16 | 0.31 |
| 1 | 1.08 | 36.00 | 0.00 | 7.40 | 9.37 | 15.10 | 139.50 | 2.50 | 5.60 | 102.60 | 0.46 | 6.23 | 12.50 | 8.50 | 4.00 | 0.71 | 0.00 | 1.25 | 26.50 | 0.18 |
| 1 | 2.70 | 217.00 | 0.00 | 10.40 | 9.52 | 17.90 | 133.50 | 3.50 | 26.20 | 98.90 | 0.56 | 8.14 | 12.70 | 11.40 | 59.00 | 10.50 | 2.00 | 6.94 | 31.80 | 0.36 |
| 1 | 2.37 | 1281.00 | 3.00 | 23.80 | 4.53 | 99.30 | 136.60 | 2.60 | 5.30 | 103.80 | 0.49 | 6.61 | 12.30 | 17.40 | 213.00 | 37.91 | 0.00 | 101.99 | 33.20 | 0.89 |
| 0 | 7.22 | 8397.00 | 27.00 | 105.70 | 6.25 | 436.90 | 136.90 | 1.20 | 3.60 | 104.00 | 0.73 | 10.94 | 13.20 | 31.70 | 475.00 | 84.55 | 6.00 | 152.55 | 28.00 | 0.53 |
| 1 | 0.02 | 3966.00 | 0.00 | 17.60 | 14.52 | 20.60 | 139.50 | 1.90 | 3.30 | 107.00 | 0.27 | 7.28 | 13.80 | 12.70 | 9.00 | 1.60 | 10.00 | 41.83 | 25.40 | 0.18 |
| 0 | 5.31 | 388.13 | 13.17 | 14.40 | 9.21 | 13.50 | 137.00 | 0.70 | 5.80 | 95.80 | 0.34 | 7.00 | 14.20 | 14.70 | 15.81 | 2.81 | 0.88 | 34.54 | 21.80 | 0.62 |
| 0 | 0.11 | 10494.00 | 73.80 | 19.40 | 8.05 | 16.50 | 141.90 | 3.60 | 5.10 | 107.20 | 0.27 | 3.58 | 13.40 | 17.90 | 0.00 | 0.00 | 52.30 | 104.50 | 21.00 | 0.00 |
| 1 | 0.15 | 627.00 | 0.00 | 12.20 | 6.12 | 19.00 | 144.30 | 1.60 | 7.20 | 109.80 | 0.35 | 7.95 | 14.00 | 15.30 | 11.00 | 1.96 | 1.00 | 0.00 | 29.70 | 0.00 |
| 1 | 1.87 | 2545.44 | 6.18 | 6.10 | 7.87 | 18.20 | 147.00 | 0.10 | 9.50 | 112.00 | 0.56 | 7.24 | 14.80 | 11.40 | 71.59 | 12.82 | 15.12 | 60.16 | 25.20 | 0.23 |
| 0 | 0.54 | 1529.77 | 22.16 | 15.90 | 4.43 | 16.80 | 139.80 | 6.80 | 10.20 | 114.00 | 0.43 | 4.38 | 14.30 | 40.80 | 350.54 | 60.47 | 37.39 | 162.52 | 16.90 | 0.63 |
| 1 | 1.87 | 85.03 | 3.51 | 16.60 | 16.45 | 18.40 | 144.00 | 9.20 | 6.10 | 109.10 | 0.61 | 6.06 | 13.50 | 18.60 | 12.79 | 2.28 | 1.75 | 44.63 | 29.40 | 0.62 |
| 0 | 0.88 | 3550.90 | 0.00 | 171.80 | 14.26 | 93.40 | 136.70 | 1.60 | 6.20 | 99.50 | 0.40 | 8.04 | 13.20 | 109.10 | 1.10 | 0.28 | 9.20 | 2.35 | 23.80 | 0.13 |
| 0 | 0.37 | 0.00 | 2.63 | 16.00 | 12.94 | 19.10 | 139.60 | 2.00 | 8.10 | 104.20 | 0.42 | 7.08 | 12.30 | 15.00 | 7.89 | 1.40 | 0.00 | 15.29 | 28.60 | 0.00 |
| 0 | 0.39 | 14.00 | 4.00 | 11.80 | 8.95 | 16.70 | 141.30 | 2.00 | 6.00 | 104.60 | 0.36 | 6.97 | 12.60 | 14.10 | 30.00 | 5.34 | 4.00 | 81.17 | 26.90 | 0.18 |
| 1 | 0.80 | 0.00 | 2.00 | 6.90 | 11.36 | 9.10 | 143.20 | 0.30 | 5.40 | 108.20 | 0.62 | 6.83 | 20.20 | 7.50 | 10.00 | 1.78 | 0.00 | 10.86 | 24.60 | 0.36 |
| 0 | 29.45 | 127.11 | 0.00 | 8.20 | 15.99 | 22.20 | 136.20 | 1.20 | 9.00 | 104.40 | 0.75 | 8.39 | 13.10 | 13.70 | 9.64 | 1.72 | 0.00 | 8.11 | 32.40 | 0.00 |
| 0 | 2.90 | 1529.77 | 22.16 | 3.40 | 8.90 | 8.90 | 140.90 | 3.20 | 12.80 | 104.30 | 0.32 | 6.67 | 15.20 | 7.90 | 350.54 | 60.47 | 37.39 | 162.52 | 26.50 | 0.63 |
| 0 | 11.80 | 42.00 | 10.00 | 5.80 | 28.24 | 13.40 | 130.40 | 0.50 | 12.50 | 96.40 | 0.85 | 10.73 | 13.60 | 8.50 | 37.00 | 6.59 | 0.00 | 73.16 | 32.30 | 0.18 |
| 0 | 11.57 | 14.00 | 10.00 | 7.60 | 15.38 | 17.00 | 129.90 | 0.90 | 10.70 | 94.90 | 0.65 | 10.83 | 12.80 | 8.10 | 27.00 | 4.81 | 0.00 | 49.31 | 38.10 | 0.18 |
| 0 | 2.22 | 198.00 | 4.00 | 5.90 | 2.39 | 14.90 | 136.20 | 2.00 | 16.60 | 101.80 | 0.04 | 4.03 | 13.00 | 9.70 | 281.00 | 50.02 | 1.00 | 22.07 | 29.60 | 0.00 |
| 0 | 0.29 | 0.00 | 0.00 | 18.60 | 9.04 | 47.42 | 137.70 | 1.60 | 9.40 | 100.70 | 0.46 | 4.43 | 11.90 | 21.00 | 10.00 | 1.78 | 1.00 | 0.00 | 26.50 | 0.00 |
| 1 | 1.87 | 2545.44 | 6.18 | 39.70 | 8.93 | 63.60 | 139.90 | 0.50 | 3.90 | 101.90 | 0.29 | 4.00 | 15.10 | 29.10 | 71.59 | 12.82 | 15.12 | 60.16 | 18.20 | 0.23 |
| 1 | 0.33 | 319.09 | 8.77 | 24.20 | 9.13 | 75.30 | 144.90 | 0.60 | 24.50 | 108.10 | 0.30 | 7.12 | 12.60 | 22.70 | 14.03 | 2.50 | 6.14 | 20.75 | 34.00 | 0.00 |
| 1 | 0.14 | 675.87 | 0.00 | 21.60 | 9.36 | 23.50 | 144.60 | 3.00 | 5.50 | 113.00 | 0.17 | 4.86 | 12.60 | 25.30 | 20.16 | 3.59 | 9.64 | 2.18 | 22.20 | 0.00 |
| 1 | 0.07 | 0.88 | 0.00 | 16.10 | 5.55 | 12.10 | 146.10 | 0.20 | 14.70 | 112.10 | 0.67 | 10.46 | 13.40 | 12.00 | 28.10 | 5.00 | 0.00 | 0.31 | 14.40 | 0.00 |
| 1 | 7.35 | 2545.44 | 6.18 | 10.80 | 7.64 | 17.90 | 139.10 | 0.00 | 9.20 | 107.50 | 0.23 | 8.86 | 12.00 | 16.40 | 71.59 | 12.82 | 15.12 | 60.16 | 24.20 | 0.23 |
| 0 | 10.80 | 50.00 | 0.00 | 83.50 | 11.58 | 252.00 | 133.70 | 0.00 | 3.50 | 98.00 | 0.57 | 7.62 | 14.10 | 114.40 | 0.00 | 0.00 | 0.00 | 9.50 | 27.70 | 0.00 |
| 0 | 8.40 | 8.00 | 0.00 | 31.00 | 9.42 | 64.60 | 130.70 | 0.00 | 10.40 | 94.00 | 0.80 | 21.25 | 12.80 | 24.90 | 1113.00 | 198.11 | 85.00 | 363.30 | 37.60 | 40.23 |
| 1 | 3.03 | 18.00 | 46.00 | 11.80 | 6.33 | 15.30 | 136.60 | 5.00 | 6.40 | 103.60 | 0.61 | 8.06 | 13.00 | 22.20 | 16.00 | 2.85 | 0.00 | 23.85 | 24.20 | 0.53 |
| 0 | 0.26 | 5.00 | 3.00 | 33.80 | 20.44 | 31.20 | 137.70 | 1.60 | 6.00 | 100.50 | 0.32 | 5.57 | 12.90 | 21.10 | 40.00 | 7.12 | 1.00 | 55.00 | 25.40 | 0.18 |
| 0 | 0.21 | 2.63 | 0.00 | 9.50 | 10.26 | 26.00 | 139.00 | 1.10 | 3.30 | 101.30 | 0.68 | 11.17 | 12.80 | 10.00 | 4.39 | 0.78 | 0.00 | 5.94 | 20.40 | 0.00 |
| 1 | 1.87 | 2731.70 | 13.60 | 8.00 | 7.54 | 14.80 | 138.30 | 0.90 | 4.70 | 102.60 | 0.31 | 6.46 | 13.30 | 11.10 | 8.90 | 2.23 | 8.80 | 68.40 | 33.00 | 0.38 |
| 1 | 1.87 | 2545.44 | 6.18 | 11.60 | 8.88 | 26.90 | 141.50 | 1.30 | 7.70 | 103.80 | 0.62 | 9.73 | 13.80 | 12.20 | 71.59 | 12.82 | 15.12 | 60.16 | 27.00 | 0.23 |
| 1 | 0.05 | 3206.80 | 11.70 | 9.40 | 8.01 | 19.80 | 135.00 | 1.60 | 10.20 | 100.10 | 0.27 | 4.48 | 13.40 | 11.20 | 2.80 | 0.70 | 14.60 | 64.90 | 26.00 | 0.47 |
| 0 | 4.37 | 6.15 | 0.00 | 56.60 | 5.56 | 30.90 | 141.10 | 2.80 | 8.60 | 103.70 | 0.61 | 10.85 | 13.60 | 38.70 | 0.00 | 0.00 | 5.27 | 0.00 | 27.00 | 0.00 |
| 0 | 0.94 | 225.29 | 0.88 | 145.40 | 5.20 | 83.10 | 133.30 | 5.50 | 5.50 | 96.00 | 0.75 | 7.21 | 13.70 | 129.90 | 116.59 | 20.75 | 0.88 | 48.53 | 30.30 | 0.31 |
| 1 | 0.24 | 8934.00 | 46.00 | 6.60 | 7.78 | 11.40 | 141.00 | 0.80 | 27.00 | 107.80 | 0.26 | 5.63 | 12.30 | 10.00 | 154.00 | 27.41 | 1.00 | 144.89 | 29.10 | 0.36 |
| 1 | 1.87 | 164.40 | 1.76 | 12.50 | 5.29 | 18.20 | 129.00 | 3.70 | 7.00 | 97.70 | 0.54 | 10.95 | 12.50 | 16.60 | 7.91 | 1.41 | 15.82 | 8.92 | 27.10 | 0.47 |
| 0 | 66.51 | 120.97 | 0.88 | 13.20 | 11.10 | 17.10 | 137.00 | 1.50 | 5.10 | 100.30 | 1.24 | 16.07 | 12.80 | 22.80 | 7.89 | 1.40 | 0.00 | 36.98 | 23.70 | 0.31 |
| 0 | 0.24 | 2394.04 | 0.00 | 30.20 | 4.60 | 169.60 | 133.70 | 0.80 | 9.00 | 103.60 | 0.37 | 7.12 | 13.10 | 24.80 | 5.26 | 0.94 | 0.88 | 2.65 | 29.60 | 0.00 |
| 1 | 1.29 | 3939.12 | 0.00 | 15.50 | 5.46 | 8.20 | 141.30 | 1.00 | 29.10 | 111.70 | 0.19 | 3.30 | 13.70 | 13.70 | 176.61 | 31.44 | 0.00 | 94.99 | 21.40 | 0.00 |
| 1 | 1.75 | 10736.00 | 23.00 | 5.90 | 7.64 | 36.70 | 138.80 | 3.50 | 7.50 | 106.00 | 0.41 | 4.72 | 15.10 | 15.00 | 231.00 | 57.75 | 51.00 | 70.00 | 23.40 | 0.25 |
| 1 | 1.87 | 2545.44 | 6.18 | 11.70 | 11.76 | 25.70 | 140.20 | 1.60 | 12.90 | 108.40 | 0.12 | 5.80 | 12.90 | 10.50 | 71.59 | 7.50 | 15.12 | 12.00 | 25.70 | 0.23 |
| 0 | 5.31 | 1529.77 | 22.16 | 24.56 | 4.98 | 47.42 | 140.00 | 12.30 | 10.30 | 108.40 | 0.70 | 6.77 | 14.00 | 25.96 | 350.54 | 60.47 | 37.39 | 162.52 | 27.54 | 0.63 |
| 1 | 0.81 | 2032.87 | 0.88 | 13.50 | 15.29 | 84.30 | 143.00 | 0.20 | 5.80 | 110.50 | 0.05 | 8.00 | 13.70 | 11.50 | 12.27 | 2.18 | 196.36 | 11.08 | 23.40 | 0.00 |
| 1 | 0.16 | 1.00 | 0.00 | 27.00 | 4.10 | 23.90 | 140.60 | 6.20 | 24.30 | 109.20 | 0.49 | 6.24 | 14.10 | 12.50 | 0.00 | 0.00 | 0.00 | 0.71 | 27.50 | 0.00 |
| 0 | 6.83 | 1378.46 | 2.64 | 8.40 | 12.25 | 22.80 | 143.30 | 1.60 | 5.70 | 105.40 | 0.79 | 9.53 | 13.90 | 11.10 | 656.70 | 116.89 | 36.92 | 151.95 | 25.50 | 0.94 |
| 0 | 1.05 | 342.00 | 51.00 | 11.90 | 4.01 | 30.10 | 141.30 | 0.70 | 15.40 | 104.50 | 0.77 | 11.81 | 13.50 | 14.20 | 44.00 | 7.83 | 1.00 | 52.15 | 33.90 | 0.18 |
| 1 | 0.41 | 2895.46 | 0.00 | 5.20 | 11.09 | 13.80 | 141.70 | 1.80 | 18.60 | 108.80 | 0.24 | 4.43 | 13.00 | 9.00 | 7.89 | 1.40 | 0.00 | 7.02 | 25.00 | 0.00 |
| 0 | 22.10 | 37.50 | 2.40 | 33.80 | 15.11 | 64.88 | 127.30 | 0.00 | 12.40 | 91.30 | 0.28 | 12.27 | 12.90 | 49.30 | 29.90 | 7.47 | 1.00 | 71.83 | 28.70 | 0.85 |
| 1 | 0.33 | 764.41 | 4.38 | 9.50 | 7.08 | 14.90 | 138.60 | 0.50 | 5.80 | 104.10 | 0.39 | 9.21 | 13.20 | 11.50 | 17.53 | 3.12 | 5.26 | 34.17 | 20.10 | 0.00 |
| 0 | 12.60 | 2368.00 | 41.00 | 22.20 | 6.19 | 119.20 | 142.30 | 0.70 | 9.00 | 106.10 | 0.80 | 18.71 | 12.90 | 11.80 | 1351.00 | 240.48 | 28.00 | 287.83 | 32.30 | 0.00 |
| 0 | 5.31 | 186.00 | 13.00 | 12.50 | 4.62 | 22.50 | 139.30 | 0.00 | 11.70 | 106.30 | 0.42 | 5.36 | 12.80 | 15.20 | 3.00 | 0.53 | 0.00 | 16.55 | 26.30 | 0.18 |
| 1 | 1.46 | 1412.23 | 0.00 | 8.00 | 4.70 | 17.90 | 142.50 | 3.30 | 7.70 | 108.80 | 0.34 | 6.06 | 16.90 | 13.20 | 8.77 | 1.56 | 2.63 | 8.43 | 19.60 | 0.00 |
| 1 | 2.88 | 163.05 | 0.00 | 11.00 | 10.74 | 20.60 | 142.60 | 0.00 | 11.10 | 104.80 | 0.46 | 10.69 | 19.10 | 10.20 | 2.63 | 0.47 | 14.90 | 1.87 | 17.50 | 0.00 |
| 1 | 0.75 | 302.43 | 0.00 | 19.60 | 11.99 | 27.90 | 139.80 | 0.20 | 11.90 | 104.70 | 0.54 | 11.00 | 14.70 | 11.20 | 1.75 | 0.31 | 1.75 | 4.06 | 15.50 | 0.00 |
| 1 | 1.31 | 28.93 | 0.00 | 22.50 | 10.82 | 27.70 | 140.40 | 0.70 | 14.50 | 98.50 | 0.62 | 10.11 | 15.40 | 10.30 | 0.00 | 0.00 | 0.88 | 0.62 | 24.70 | 0.00 |
| 1 | 1.53 | 213.00 | 0.00 | 14.00 | 4.40 | 17.50 | 140.10 | 1.50 | 7.60 | 102.20 | 0.55 | 8.26 | 16.90 | 11.30 | 0.00 | 0.00 | 0.00 | 1.42 | 20.40 | 0.00 |
| 1 | 1.46 | 500.00 | 0.00 | 15.20 | 4.37 | 21.60 | 137.70 | 2.60 | 17.00 | 105.40 | 0.33 | 4.78 | 13.40 | 15.30 | 0.00 | 0.00 | 0.00 | 33.00 | 33.90 | 0.00 |
| 1 | 1.55 | 2717.51 | 0.00 | 6.90 | 6.81 | 16.10 | 144.90 | 2.40 | 20.40 | 109.60 | 0.54 | 7.58 | 14.10 | 10.40 | 0.00 | 0.00 | 1.75 | 1.87 | 19.90 | 0.00 |
| 0 | 0.16 | 44.71 | 20.16 | 14.60 | 11.03 | 24.10 | 139.30 | 4.90 | 6.70 | 102.90 | 0.35 | 5.16 | 12.70 | 17.70 | 2.63 | 0.47 | 0.00 | 35.58 | 36.10 | 0.00 |
| 1 | 1.09 | 8000.00 | 59.00 | 8.80 | 8.41 | 17.80 | 141.40 | 2.20 | 6.20 | 109.00 | 0.34 | 4.67 | 13.40 | 14.00 | 14.00 | 3.50 | 0.00 | 339.50 | 31.50 | 0.00 |
| 0 | 2.02 | 6256.30 | 17.70 | 11.90 | 7.22 | 14.60 | 139.00 | 0.70 | 4.80 | 107.70 | 0.21 | 3.33 | 13.10 | 20.90 | 5.60 | 1.40 | 0.00 | 216.28 | 24.30 | 1.05 |
| 1 | 0.35 | 935.19 | 7.90 | 15.30 | 7.47 | 15.40 | 142.60 | 1.80 | 5.90 | 106.40 | 0.38 | 5.90 | 12.60 | 15.50 | 18.44 | 3.28 | 12.29 | 28.15 | 30.30 | 0.16 |
| 1 | 0.12 | 8000.00 | 0.00 | 22.70 | 6.84 | 27.20 | 140.20 | 0.70 | 8.20 | 104.60 | 0.48 | 8.42 | 12.80 | 18.40 | 4.00 | 1.00 | 0.00 | 5.50 | 26.60 | 0.00 |
| 1 | 1.96 | 2545.44 | 6.18 | 18.10 | 5.19 | 34.70 | 148.60 | 1.50 | 9.10 | 111.40 | 0.30 | 4.20 | 20.30 | 26.70 | 71.59 | 12.82 | 15.12 | 60.16 | 16.40 | 0.23 |
| 0 | 5.31 | 71.50 | 15.60 | 24.56 | 9.39 | 47.42 | 143.10 | 1.40 | 8.78 | 108.80 | 0.40 | 8.19 | 14.90 | 25.96 | 29.40 | 7.35 | 0.00 | 69.15 | 27.54 | 0.13 |
| 0 | 21.20 | 50.00 | 0.00 | 77.60 | 7.54 | 159.00 | 124.50 | 0.00 | 10.20 | 92.60 | 1.77 | 21.85 | 13.90 | 54.30 | 23.00 | 5.75 | 0.00 | 71.75 | 29.90 | 0.00 |
| 0 | 2.07 | 0.00 | 0.00 | 26.70 | 5.25 | 68.70 | 138.10 | 1.20 | 4.50 | 106.20 | 0.45 | 6.99 | 13.20 | 15.30 | 14.00 | 3.50 | 0.00 | 12.75 | 37.20 | 0.00 |
| 0 | 0.84 | 1529.77 | 22.16 | 14.80 | 5.84 | 57.10 | 138.20 | 1.50 | 6.40 | 106.50 | 0.63 | 6.68 | 15.60 | 14.70 | 350.54 | 60.47 | 37.39 | 162.52 | 42.00 | 0.63 |
| 0 | 0.36 | 2217.90 | 6.00 | 24.56 | 11.96 | 47.42 | 126.80 | 1.20 | 8.78 | 94.60 | 0.45 | 7.17 | 13.90 | 25.96 | 5.70 | 1.43 | 0.00 | 80.42 | 27.54 | 0.15 |
| 0 | 2.04 | 1529.77 | 22.16 | 7.90 | 13.54 | 16.30 | 123.20 | 0.00 | 6.40 | 89.80 | 0.51 | 13.03 | 14.70 | 9.90 | 350.54 | 60.47 | 37.39 | 162.52 | 26.10 | 0.63 |
| 0 | 19.40 | 444.44 | 1.75 | 70.20 | 5.92 | 117.30 | 133.60 | 0.30 | 10.10 | 104.20 | 0.38 | 10.34 | 15.90 | 79.10 | 210.39 | 37.45 | 345.39 | 10.14 | 31.40 | 0.00 |
| 1 | 1.87 | 1116.97 | 1.76 | 27.00 | 8.33 | 28.76 | 142.30 | 1.50 | 4.50 | 106.00 | 0.57 | 7.40 | 13.60 | 35.00 | 2.01 | 0.36 | 0.00 | 175.53 | 26.20 | 0.00 |
| 0 | 5.78 | 412.00 | 0.00 | 12.80 | 9.39 | 17.60 | 131.90 | 0.30 | 3.30 | 102.20 | 0.50 | 8.44 | 12.30 | 19.40 | 28.00 | 4.98 | 4.00 | 10.68 | 29.60 | 0.00 |
| 0 | 2.17 | 1073.00 | 0.00 | 18.70 | 5.85 | 23.60 | 135.90 | 1.30 | 8.40 | 100.00 | 0.22 | 3.44 | 13.70 | 26.00 | 54.00 | 13.50 | 6.00 | 1.25 | 28.70 | 0.25 |
| 1 | 0.66 | 1.75 | 0.00 | 3.90 | 4.53 | 11.00 | 148.20 | 2.20 | 48.20 | 107.80 | 0.28 | 7.36 | 13.40 | 5.20 | 3.51 | 0.62 | 0.00 | 2.50 | 24.70 | 0.00 |
| 0 | 0.33 | 18.41 | 0.00 | 24.00 | 15.18 | 22.30 | 141.80 | 0.30 | 5.90 | 102.80 | 0.25 | 5.71 | 11.80 | 17.50 | 114.84 | 20.44 | 11.40 | 16.38 | 31.50 | 0.00 |
| 0 | 0.97 | 15.78 | 0.00 | 16.90 | 6.17 | 21.50 | 146.80 | 1.00 | 1.80 | 102.20 | 0.10 | 6.95 | 13.30 | 32.00 | 6.14 | 1.09 | 2.63 | 0.94 | 33.20 | 0.00 |
| 1 | 1.12 | 123.60 | 0.00 | 12.50 | 4.52 | 16.30 | 137.60 | 3.80 | 3.70 | 105.70 | 0.31 | 5.24 | 11.20 | 17.20 | 1.75 | 0.31 | 3.51 | 4.37 | 20.40 | 0.00 |
| 1 | 4.03 | 50.00 | 0.00 | 6.40 | 6.31 | 28.50 | 130.20 | 1.10 | 7.30 | 96.50 | 0.67 | 6.93 | 13.40 | 11.50 | 0.00 | 0.00 | 5.00 | 42.25 | 28.40 | 0.00 |
| 1 | 1.87 | 2545.44 | 6.18 | 20.40 | 14.03 | 32.30 | 132.40 | 0.00 | 11.70 | 94.40 | 1.59 | 11.94 | 14.10 | 18.90 | 71.59 | 12.82 | 15.12 | 60.16 | 26.00 | 0.23 |
| 0 | 5.31 | 2948.80 | 3.40 | 17.20 | 3.79 | 55.60 | 138.00 | 9.60 | 10.70 | 105.10 | 0.51 | 6.23 | 13.50 | 12.80 | 0.00 | 0.00 | 0.50 | 7.88 | 23.00 | 0.13 |
| 0 | 0.31 | 244.00 | 0.00 | 28.00 | 6.72 | 29.60 | 137.80 | 0.50 | 4.80 | 100.70 | 0.50 | 6.67 | 11.50 | 33.10 | 8.00 | 1.42 | 0.00 | 0.18 | 33.20 | 0.18 |
| 1 | 0.60 | 1947.25 | 4.40 | 8.60 | 7.64 | 13.80 | 138.00 | 1.90 | 25.60 | 104.80 | 1.00 | 11.04 | 17.80 | 25.60 | 7.03 | 1.25 | 0.00 | 28.95 | 30.70 | 0.16 |
| 0 | 7.89 | 1536.71 | 31.61 | 28.00 | 6.81 | 21.70 | 134.80 | 1.20 | 4.40 | 104.10 | 0.44 | 4.80 | 12.60 | 33.60 | 229.19 | 40.80 | 64.10 | 109.60 | 18.50 | 0.00 |
| 0 | 5.31 | 193.19 | 0.00 | 11.80 | 13.40 | 15.90 | 137.70 | 1.60 | 8.90 | 99.70 | 0.39 | 5.40 | 12.60 | 11.20 | 2.63 | 0.47 | 20.20 | 0.94 | 22.60 | 0.16 |
| 0 | 8.66 | 22.90 | 0.00 | 320.00 | 12.15 | 263.00 | 128.70 | 2.20 | 10.60 | 95.60 | 0.77 | 15.79 | 13.20 | 213.00 | 1.00 | 0.25 | 0.00 | 0.23 | 30.80 | 0.00 |
| 1 | 3.27 | 1378.00 | 0.00 | 7.40 | 6.16 | 35.40 | 139.90 | 0.20 | 7.40 | 100.70 | 0.18 | 2.12 | 16.80 | 16.30 | 331.00 | 58.92 | 1.00 | 132.79 | 28.40 | 1.07 |
| 0 | 0.12 | 139.38 | 6.14 | 7.50 | 6.31 | 18.30 | 141.00 | 4.10 | 7.30 | 107.90 | 0.26 | 5.66 | 13.50 | 11.90 | 5.26 | 0.94 | 0.88 | 19.04 | 24.60 | 0.00 |
| 0 | 5.31 | 1529.77 | 22.16 | 39.70 | 9.24 | 31.50 | 143.50 | 3.90 | 8.10 | 101.00 | 0.45 | 6.70 | 13.80 | 41.60 | 350.54 | 0.00 | 37.39 | 7.00 | 34.80 | 0.63 |
| 0 | 0.41 | 397.98 | 0.00 | 21.20 | 27.82 | 34.50 | 134.00 | 2.50 | 6.70 | 98.20 | 0.42 | 6.74 | 12.80 | 19.80 | 121.85 | 21.69 | 0.00 | 406.01 | 31.90 | 0.16 |
| 0 | 0.14 | 11.40 | 15.78 | 15.60 | 9.36 | 24.70 | 143.60 | 1.90 | 10.10 | 108.30 | 0.40 | 6.40 | 13.00 | 19.60 | 0.00 | 0.00 | 2.63 | 46.50 | 25.80 | 0.31 |
| 0 | 0.11 | 6755.00 | 32.00 | 12.50 | 6.71 | 17.00 | 142.70 | 5.00 | 6.00 | 107.90 | 0.37 | 7.76 | 12.80 | 14.80 | 31.00 | 5.52 | 1.00 | 97.19 | 23.40 | 0.18 |
| 0 | 1.73 | 446.00 | 13.00 | 32.30 | 8.82 | 131.10 | 140.60 | 1.00 | 16.20 | 105.30 | 0.62 | 7.47 | 13.40 | 39.40 | 30.00 | 5.34 | 3.00 | 25.63 | 28.10 | 0.53 |
| 0 | 0.30 | 8688.10 | 30.50 | 3.90 | 5.02 | 16.60 | 141.40 | 3.00 | 14.90 | 105.50 | 0.49 | 5.67 | 13.50 | 16.30 | 20.10 | 5.03 | 3.80 | 357.18 | 27.90 | 2.23 |
| 0 | 2.46 | 864.70 | 0.00 | 12.00 | 6.32 | 47.42 | 130.10 | 0.20 | 4.90 | 97.90 | 0.82 | 10.75 | 20.30 | 14.00 | 1605.44 | 285.77 | 39.30 | 2370.64 | 29.60 | 13.46 |
| 0 | 0.18 | 1211.48 | 3.51 | 21.50 | 8.08 | 15.50 | 143.80 | 1.40 | 5.10 | 103.40 | 0.46 | 5.76 | 13.90 | 21.50 | 43.83 | 7.80 | 0.00 | 164.15 | 28.70 | 0.47 |
| 1 | 0.08 | 1682.23 | 3.51 | 19.90 | 6.51 | 14.70 | 142.30 | 4.70 | 8.60 | 105.90 | 0.47 | 5.33 | 13.70 | 21.50 | 15.78 | 2.81 | 93.80 | 53.83 | 24.50 | 0.31 |
| 1 | 0.29 | 412.89 | 0.00 | 24.00 | 5.43 | 28.76 | 142.80 | 2.10 | 3.60 | 102.10 | 0.46 | 8.55 | 12.90 | 34.00 | 17.00 | 3.03 | 0.88 | 65.07 | 32.80 | 0.62 |
| 1 | 1.87 | 1422.55 | 63.22 | 7.10 | 7.64 | 26.80 | 143.00 | 1.30 | 24.40 | 105.40 | 0.56 | 8.50 | 13.60 | 14.50 | 188.80 | 33.61 | 0.00 | 419.99 | 20.60 | 0.31 |
| 1 | 0.41 | 73.00 | 3.00 | 56.00 | 8.65 | 42.50 | 140.90 | 4.00 | 11.30 | 108.70 | 0.37 | 6.44 | 14.00 | 38.60 | 17.00 | 3.03 | 0.00 | 53.58 | 26.80 | 0.18 |
| 0 | 0.33 | 4.00 | 0.00 | 23.40 | 6.26 | 23.20 | 145.00 | 2.70 | 4.50 | 110.00 | 0.19 | 4.03 | 14.20 | 15.30 | 224.00 | 39.87 | 44.00 | 7.30 | 19.70 | 0.53 |
| 1 | 0.14 | 110.45 | 1.75 | 12.60 | 13.31 | 15.50 | 140.00 | 2.70 | 8.30 | 103.30 | 0.52 | 8.72 | 13.60 | 14.80 | 11.20 | 1.99 | 4.38 | 71.15 | 27.80 | 0.16 |
| 0 | 1.39 | 3.00 | 0.00 | 9.20 | 6.99 | 13.60 | 139.20 | 3.80 | 8.90 | 107.60 | 0.52 | 8.22 | 14.00 | 15.50 | 1.00 | 0.18 | 0.00 | 0.18 | 28.70 | 0.00 |
| 0 | 1.42 | 1529.77 | 22.16 | 6.20 | 5.45 | 33.40 | 138.70 | 0.40 | 3.60 | 108.30 | 0.08 | 6.77 | 19.60 | 16.30 | 350.54 | 60.47 | 37.39 | 162.52 | 31.40 | 0.63 |
| 1 | 0.31 | 187.60 | 7.89 | 6.10 | 3.53 | 19.50 | 141.00 | 4.00 | 17.60 | 113.90 | 0.52 | 8.45 | 12.20 | 10.80 | 16.07 | 2.86 | 0.00 | 83.95 | 26.90 | 0.00 |
| 0 | 0.43 | 124.84 | 0.00 | 14.60 | 14.49 | 19.30 | 128.90 | 1.90 | 3.60 | 92.30 | 0.51 | 5.17 | 12.50 | 13.40 | 7.03 | 1.25 | 53.63 | 73.70 | 31.60 | 0.31 |
| 0 | 31.85 | 590.60 | 0.00 | 38.40 | 9.39 | 47.42 | 138.40 | 0.10 | 23.10 | 108.00 | 0.81 | 13.85 | 13.80 | 84.30 | 153.89 | 27.39 | 2969.62 | 169.53 | 29.00 | 3.70 |
| 1 | 2.66 | 5910.00 | 150.00 | 13.40 | 4.89 | 20.40 | 138.70 | 3.10 | 12.00 | 106.40 | 0.37 | 7.25 | 17.20 | 18.90 | 2777.00 | 494.31 | 35.00 | 702.57 | 21.00 | 0.36 |
| 0 | 5.31 | 1529.77 | 22.16 | 10.60 | 7.32 | 20.90 | 143.40 | 2.50 | 4.80 | 106.90 | 0.40 | 7.40 | 13.00 | 15.70 | 350.54 | 0.00 | 37.39 | 0.00 | 35.50 | 0.63 |
| 1 | 7.41 | 328.10 | 0.00 | 8.50 | 13.32 | 20.30 | 124.50 | 1.40 | 7.90 | 90.10 | 0.42 | 8.08 | 14.50 | 12.50 | 18.50 | 4.63 | 1.50 | 5.58 | 26.70 | 0.00 |
| 1 | 1.87 | 3367.70 | 3.50 | 10.30 | 7.64 | 23.90 | 125.90 | 2.60 | 9.60 | 89.70 | 0.46 | 8.03 | 14.30 | 14.00 | 1.70 | 0.42 | 3.50 | 114.42 | 29.80 | 0.53 |
| 1 | 1.87 | 2545.44 | 6.18 | 26.10 | 7.64 | 27.90 | 143.40 | 0.20 | 10.60 | 105.90 | 0.36 | 5.60 | 12.60 | 15.50 | 71.59 | 12.82 | 15.12 | 60.16 | 18.90 | 0.23 |
| 0 | 0.44 | 149.00 | 0.00 | 14.30 | 14.00 | 20.50 | 138.50 | 1.20 | 8.80 | 103.80 | 0.57 | 9.14 | 13.10 | 18.00 | 43.00 | 7.65 | 0.00 | 32.22 | 33.90 | 0.53 |
| 1 | 1.01 | 6312.14 | 0.00 | 11.90 | 10.24 | 25.20 | 139.90 | 0.80 | 8.50 | 98.70 | 0.46 | 6.55 | 12.80 | 12.10 | 33.90 | 6.03 | 2.61 | 416.84 | 28.10 | 0.93 |
| 1 | 1.87 | 7837.00 | 9.00 | 10.00 | 5.44 | 13.20 | 140.20 | 2.30 | 4.10 | 107.10 | 0.50 | 4.75 | 21.10 | 18.40 | 57.00 | 10.15 | 40.00 | 43.97 | 24.70 | 0.18 |
| 0 | 0.59 | 2161.00 | 0.00 | 10.60 | 8.45 | 23.20 | 118.90 | 0.40 | 4.10 | 85.10 | 0.62 | 3.47 | 15.30 | 11.90 | 1040.00 | 185.12 | 0.00 | 6.41 | 28.10 | 0.00 |
| 0 | 0.41 | 513.00 | 0.00 | 20.60 | 10.39 | 25.70 | 144.80 | 0.60 | 7.00 | 106.90 | 0.39 | 7.19 | 12.60 | 18.90 | 4.00 | 0.71 | 0.00 | 2.31 | 39.00 | 0.00 |
| 0 | 14.80 | 2280.00 | 23.00 | 8.90 | 16.82 | 13.90 | 128.10 | 0.10 | 6.30 | 91.50 | 0.23 | 12.49 | 13.10 | 12.60 | 136.00 | 24.21 | 8.00 | 174.97 | 22.60 | 0.53 |
| 1 | 0.30 | 8000.00 | 0.00 | 9.50 | 4.61 | 14.90 | 127.20 | 5.60 | 10.00 | 103.00 | 0.41 | 3.42 | 13.80 | 11.40 | 0.00 | 0.00 | 0.00 | 100.75 | 23.50 | 0.00 |
| 0 | 7.42 | 50.00 | 0.00 | 15.50 | 23.44 | 15.00 | 132.90 | 0.10 | 4.90 | 106.10 | 0.44 | 7.11 | 13.30 | 16.40 | 10.00 | 2.50 | 80.00 | 82.75 | 21.20 | 0.50 |
| 0 | 1.08 | 1529.77 | 22.16 | 12.30 | 27.32 | 20.30 | 135.00 | 1.10 | 6.90 | 99.60 | 0.36 | 6.90 | 12.50 | 17.10 | 350.54 | 60.47 | 37.39 | 162.52 | 21.50 | 0.63 |
| 1 | 1.87 | 2545.44 | 6.18 | 12.60 | 7.81 | 15.40 | 142.60 | 2.50 | 5.90 | 107.50 | 0.17 | 4.70 | 13.10 | 15.30 | 71.59 | 12.82 | 15.12 | 60.16 | 20.40 | 0.23 |
| 0 | 0.94 | 114.00 | 62.00 | 10.90 | 10.21 | 29.50 | 141.40 | 1.80 | 6.20 | 105.50 | 0.30 | 6.81 | 13.20 | 12.10 | 227.00 | 40.41 | 0.00 | 664.30 | 35.60 | 0.36 |
| 1 | 1.87 | 20.20 | 13.17 | 23.10 | 6.51 | 28.60 | 137.70 | 1.90 | 7.40 | 101.90 | 0.33 | 6.20 | 12.30 | 25.70 | 65.86 | 11.72 | 0.00 | 76.28 | 25.90 | 0.31 |
| 1 | 2.69 | 135.00 | 29.00 | 19.90 | 12.46 | 109.60 | 133.40 | 3.20 | 21.60 | 106.60 | 0.55 | 9.05 | 15.00 | 18.70 | 24.00 | 4.27 | 0.00 | 152.90 | 42.50 | 0.36 |
| 1 | 6.38 | 11271.00 | 97.00 | 9.80 | 8.08 | 28.76 | 130.70 | 0.20 | 36.80 | 100.80 | 0.28 | 9.74 | 14.20 | 9.10 | 275.00 | 48.95 | 3.00 | 441.80 | 30.30 | 1.25 |
| 0 | 5.31 | 494.07 | 0.00 | 5.70 | 14.23 | 39.90 | 141.50 | 0.10 | 10.40 | 105.30 | 0.63 | 13.36 | 12.90 | 8.10 | 123.96 | 22.06 | 237.36 | 78.24 | 26.30 | 0.94 |
| 0 | 0.50 | 1071.23 | 0.00 | 10.00 | 16.49 | 37.60 | 141.00 | 1.80 | 6.50 | 99.60 | 0.45 | 10.76 | 13.30 | 19.00 | 159.54 | 28.40 | 35.94 | 507.75 | 31.80 | 1.09 |
| 0 | 0.12 | 944.99 | 0.00 | 12.80 | 4.28 | 28.30 | 143.70 | 3.00 | 9.90 | 104.50 | 0.62 | 9.19 | 14.80 | 14.20 | 141.14 | 25.12 | 128.86 | 328.61 | 25.20 | 0.62 |
| 0 | 0.17 | 175.32 | 0.88 | 13.10 | 6.42 | 18.70 | 142.70 | 3.00 | 6.00 | 107.10 | 0.31 | 4.77 | 12.20 | 13.40 | 5.26 | 0.94 | 0.00 | 10.77 | 24.10 | 0.00 |
| 1 | 1.22 | 1133.00 | 0.00 | 5.50 | 4.71 | 18.00 | 138.30 | 1.20 | 11.80 | 101.10 | 0.13 | 2.80 | 14.20 | 9.90 | 4.00 | 1.00 | 3.00 | 9.75 | 26.30 | 0.00 |
| 0 | 1.34 | 94.07 | 1.76 | 12.00 | 16.40 | 47.42 | 137.40 | 0.30 | 5.80 | 101.50 | 0.31 | 6.97 | 12.50 | 11.00 | 13.19 | 2.35 | 7.03 | 10.48 | 27.90 | 0.16 |
| 0 | 0.10 | 8000.00 | 0.00 | 5.40 | 9.04 | 41.80 | 138.90 | 1.90 | 6.30 | 101.70 | 0.39 | 5.99 | 13.30 | 11.20 | 16.00 | 4.00 | 20.00 | 35.25 | 26.80 | 0.00 |
| 0 | 0.32 | 1334.21 | 0.00 | 15.50 | 3.56 | 21.20 | 144.50 | 2.80 | 4.70 | 103.30 | 0.32 | 5.96 | 13.70 | 12.40 | 3.51 | 0.62 | 3.51 | 1.87 | 29.20 | 0.16 |
| 1 | 1.87 | 1644.00 | 0.00 | 5.70 | 10.78 | 12.20 | 142.40 | 3.60 | 4.10 | 104.10 | 0.40 | 9.30 | 14.90 | 14.00 | 12.00 | 2.14 | 6.00 | 6.94 | 25.10 | 0.00 |
| 0 | 4.92 | 500.00 | 121.00 | 12.80 | 15.82 | 20.80 | 135.40 | 0.70 | 22.80 | 106.60 | 0.57 | 11.67 | 14.20 | 15.80 | 139.00 | 34.75 | 0.00 | 422.50 | 24.90 | 0.00 |
| 1 | 3.88 | 1188.97 | 0.00 | 12.10 | 5.00 | 84.00 | 138.60 | 1.60 | 11.40 | 100.80 | 0.64 | 8.90 | 14.70 | 15.50 | 128.21 | 22.82 | 0.00 | 469.38 | 23.40 | 0.31 |
| 0 | 15.30 | 1972.25 | 6.15 | 21.00 | 8.90 | 25.40 | 135.00 | 0.00 | 4.50 | 101.90 | 0.74 | 11.50 | 14.80 | 16.00 | 149.28 | 26.57 | 0.88 | 123.01 | 27.60 | 0.31 |
| 1 | 0.26 | 2071.11 | 0.00 | 7.20 | 3.46 | 11.40 | 142.10 | 5.00 | 26.20 | 111.50 | 0.30 | 6.24 | 12.70 | 12.00 | 146.47 | 26.07 | 159.87 | 118.75 | 23.40 | 0.64 |
| 1 | 6.04 | 2582.00 | 13.00 | 180.50 | 10.16 | 63.30 | 131.50 | 0.70 | 25.40 | 90.80 | 0.39 | 10.23 | 17.00 | 24.40 | 191.00 | 34.00 | 17.00 | 103.42 | 30.90 | 0.00 |
| 1 | 1.16 | 15.81 | 0.00 | 15.00 | 7.64 | 28.76 | 144.40 | 0.70 | 4.40 | 102.30 | 0.22 | 4.30 | 12.30 | 11.00 | 97.47 | 17.35 | 0.88 | 315.27 | 31.90 | 0.78 |
| 0 | 5.62 | 11540.40 | 2.80 | 10.80 | 10.49 | 30.40 | 137.90 | 8.40 | 5.20 | 99.70 | 0.61 | 6.51 | 13.60 | 19.50 | 0.00 | 0.00 | 0.00 | 16.57 | 28.90 | 0.35 |
| 1 | 0.08 | 8.77 | 0.00 | 21.30 | 6.90 | 13.30 | 145.00 | 1.90 | 4.20 | 110.10 | 0.23 | 4.13 | 12.80 | 22.90 | 0.88 | 0.16 | 0.88 | 1.40 | 19.90 | 0.00 |
| 1 | 0.03 | 45.58 | 0.00 | 21.00 | 6.20 | 14.80 | 145.50 | 2.00 | 3.40 | 109.50 | 0.24 | 4.30 | 12.70 | 20.60 | 2.63 | 0.47 | 0.88 | 7.96 | 19.10 | 0.00 |
| 1 | 0.17 | 95.55 | 0.00 | 15.80 | 4.81 | 13.10 | 146.50 | 1.60 | 4.30 | 107.90 | 0.28 | 5.49 | 12.60 | 18.90 | 0.00 | 0.00 | 3.51 | 9.05 | 21.50 | 0.00 |
| 1 | 11.73 | 501.42 | 0.88 | 22.90 | 5.08 | 67.20 | 142.00 | 2.10 | 7.10 | 107.10 | 0.65 | 7.89 | 14.40 | 19.60 | 7.01 | 1.25 | 3.51 | 10.61 | 22.50 | 0.62 |
| 0 | 2.67 | 5.00 | 11.00 | 12.90 | 6.43 | 19.90 | 131.40 | 2.10 | 5.10 | 94.00 | 0.38 | 4.17 | 12.10 | 22.40 | 698.00 | 124.24 | 0.00 | 198.83 | 15.30 | 0.18 |
| 1 | 0.25 | 2528.16 | 2.63 | 16.00 | 11.23 | 16.60 | 139.90 | 7.60 | 8.20 | 105.10 | 0.45 | 9.82 | 12.60 | 15.00 | 11.40 | 2.03 | 6.14 | 20.13 | 33.00 | 0.16 |
| 0 | 12.10 | 24.00 | 1.00 | 25.50 | 5.07 | 27.10 | 139.60 | 0.80 | 2.10 | 101.90 | 0.92 | 18.51 | 13.50 | 25.80 | 46.00 | 8.19 | 2.00 | 8.37 | 30.20 | 0.00 |
| 0 | 2.00 | 204.00 | 9.00 | 9.40 | 3.85 | 24.50 | 134.30 | 1.40 | 3.80 | 103.50 | 0.70 | 9.55 | 17.30 | 18.00 | 65.00 | 11.57 | 0.00 | 28.66 | 32.00 | 0.18 |
| 0 | 0.65 | 308.22 | 0.88 | 24.56 | 6.55 | 47.42 | 138.20 | 2.80 | 5.60 | 103.60 | 0.39 | 4.60 | 13.60 | 25.96 | 22.83 | 4.06 | 0.00 | 68.77 | 27.54 | 0.00 |
| 0 | 9.55 | 11.40 | 3.51 | 24.80 | 7.38 | 193.60 | 142.50 | 1.60 | 4.30 | 106.40 | 0.49 | 5.82 | 13.70 | 17.30 | 21.92 | 3.90 | 1.75 | 23.09 | 33.70 | 0.00 |
| 0 | 0.08 | 42.20 | 14.07 | 22.70 | 5.47 | 17.90 | 143.40 | 5.40 | 6.20 | 106.80 | 0.45 | 6.62 | 13.10 | 23.00 | 19.34 | 3.44 | 0.00 | 80.90 | 27.80 | 0.16 |
| 0 | 6.39 | 277.00 | 2.00 | 57.50 | 8.64 | 9.70 | 144.90 | 0.40 | 4.40 | 112.30 | 0.64 | 7.18 | 13.20 | 21.60 | 73.00 | 12.99 | 6.00 | 38.80 | 21.60 | 0.00 |
| 0 | 3.86 | 500.00 | 88.00 | 17.40 | 10.54 | 64.40 | 140.10 | 1.80 | 8.40 | 104.40 | 0.32 | 4.84 | 12.50 | 13.90 | 17.00 | 4.25 | 0.00 | 956.50 | 24.30 | 0.00 |
| 1 | 0.31 | 12.27 | 0.00 | 7.40 | 3.97 | 15.60 | 140.50 | 2.60 | 8.80 | 110.50 | 0.37 | 6.25 | 14.10 | 16.40 | 0.00 | 0.00 | 2.63 | 1.25 | 21.60 | 0.16 |
| 1 | 2.59 | 924.00 | 0.00 | 7.40 | 6.49 | 27.60 | 139.90 | 1.00 | 12.60 | 108.50 | 0.39 | 6.42 | 12.50 | 10.30 | 329.00 | 58.56 | 0.00 | 21.89 | 26.00 | 0.53 |
| 0 | 1.40 | 0.00 | 0.00 | 34.80 | 7.46 | 247.10 | 133.70 | 0.30 | 3.80 | 100.60 | 0.49 | 9.97 | 12.60 | 48.80 | 3.00 | 0.75 | 20.00 | 0.25 | 24.60 | 0.00 |
| 1 | 0.31 | 6620.00 | 7.00 | 13.70 | 12.80 | 118.00 | 140.30 | 3.10 | 5.00 | 107.40 | 0.44 | 7.69 | 13.80 | 20.80 | 34.00 | 6.05 | 5.00 | 32.75 | 37.10 | 0.00 |
| 0 | 0.17 | 0.00 | 0.00 | 54.00 | 10.26 | 47.42 | 136.40 | 3.40 | 3.30 | 98.90 | 0.37 | 6.51 | 14.00 | 22.00 | 4.38 | 0.78 | 0.00 | 15.29 | 22.60 | 0.00 |
| 0 | 0.99 | 857.04 | 0.00 | 10.90 | 12.05 | 19.50 | 140.00 | 1.40 | 17.60 | 93.40 | 0.49 | 9.90 | 12.70 | 11.80 | 8.83 | 1.57 | 0.00 | 57.83 | 20.00 | 0.00 |
| 0 | 0.19 | 1207.91 | 3.52 | 11.10 | 10.27 | 18.20 | 140.50 | 1.90 | 6.50 | 104.50 | 0.26 | 4.45 | 12.10 | 11.80 | 21.10 | 3.76 | 0.88 | 29.89 | 21.30 | 0.00 |
| 1 | 0.87 | 283.15 | 0.00 | 7.10 | 10.46 | 22.40 | 133.40 | 1.50 | 19.20 | 96.00 | 0.46 | 6.57 | 12.50 | 10.90 | 15.78 | 2.81 | 0.88 | 5.93 | 24.60 | 0.16 |
| 0 | 5.45 | 1115.06 | 10.52 | 13.80 | 11.20 | 86.40 | 140.00 | 1.00 | 7.70 | 98.10 | 0.48 | 9.44 | 13.50 | 15.50 | 20.81 | 3.70 | 4.38 | 97.84 | 32.80 | 0.31 |
| 0 | 20.40 | 8000.00 | 0.00 | 13.10 | 10.72 | 15.30 | 138.70 | 0.00 | 7.90 | 108.20 | 0.90 | 19.28 | 13.90 | 17.10 | 0.00 | 0.00 | 0.00 | 14.50 | 25.30 | 0.00 |
| 0 | 4.83 | 288.00 | 0.00 | 52.00 | 5.59 | 24.40 | 144.70 | 0.60 | 14.50 | 106.60 | 0.47 | 4.06 | 12.90 | 49.10 | 6.00 | 1.07 | 5.00 | 0.71 | 26.90 | 0.00 |
| 0 | 2.18 | 74.00 | 0.00 | 7.30 | 5.42 | 19.50 | 118.60 | 1.00 | 16.90 | 87.30 | 0.86 | 8.97 | 15.20 | 12.90 | 7.00 | 1.25 | 0.00 | 7.12 | 19.50 | 0.00 |
| 0 | 1.66 | 3.52 | 15.82 | 12.50 | 9.39 | 66.40 | 139.30 | 2.00 | 4.90 | 105.80 | 0.34 | 5.44 | 14.70 | 14.10 | 62.42 | 11.11 | 0.00 | 140.52 | 36.70 | 0.00 |
| 0 | 10.30 | 50.00 | 0.00 | 31.20 | 13.45 | 41.50 | 133.20 | 1.10 | 6.60 | 100.50 | 0.28 | 5.64 | 12.10 | 16.10 | 12.00 | 3.00 | 0.00 | 18.25 | 19.80 | 0.00 |
| 1 | 0.36 | 10809.00 | 0.00 | 12.50 | 12.87 | 24.40 | 138.90 | 2.90 | 16.30 | 106.80 | 0.30 | 6.51 | 14.90 | 15.10 | 13.00 | 2.31 | 0.00 | 6.76 | 19.40 | 0.00 |
| 0 | 5.31 | 1529.77 | 22.16 | 9.50 | 5.49 | 67.20 | 136.70 | 0.10 | 2.40 | 102.50 | 0.80 | 13.47 | 20.00 | 20.60 | 350.54 | 60.47 | 37.39 | 162.52 | 28.70 | 0.63 |
| 0 | 0.09 | 1019.00 | 3.00 | 13.20 | 4.59 | 17.80 | 145.00 | 3.10 | 6.50 | 110.70 | 0.47 | 7.94 | 13.70 | 12.50 | 47.00 | 8.37 | 0.00 | 37.91 | 25.30 | 0.36 |
| 0 | 1.19 | 2411.00 | 18.00 | 14.30 | 6.60 | 39.70 | 142.60 | 4.20 | 4.70 | 106.80 | 0.59 | 8.17 | 13.30 | 25.60 | 110.00 | 19.58 | 0.00 | 37.91 | 30.10 | 0.00 |
| 0 | 5.31 | 1529.77 | 22.16 | 64.60 | 6.67 | 48.70 | 140.70 | 0.80 | 8.90 | 104.40 | 0.62 | 7.87 | 14.00 | 36.20 | 350.54 | 60.47 | 37.39 | 162.52 | 19.60 | 0.63 |
| 1 | 0.04 | 14755.30 | 0.00 | 14.10 | 10.03 | 24.40 | 140.30 | 1.10 | 5.60 | 102.40 | 0.38 | 6.42 | 12.60 | 14.00 | 0.00 | 0.00 | 4.90 | 5.05 | 28.10 | 0.00 |
| 0 | 7.30 | 1529.77 | 22.16 | 13.60 | 6.26 | 18.20 | 140.00 | 0.20 | 6.00 | 108.30 | 0.28 | 9.50 | 14.40 | 12.70 | 350.54 | 0.00 | 37.39 | 4.00 | 18.40 | 0.63 |
| 0 | 8.00 | 9046.86 | 17.58 | 12.10 | 7.64 | 31.50 | 138.80 | 0.80 | 17.20 | 103.00 | 0.70 | 6.18 | 16.10 | 9.40 | 305.90 | 54.45 | 2.34 | 155.42 | 25.40 | 0.21 |
| 0 | 0.30 | 1428.01 | 0.00 | 17.50 | 7.34 | 28.20 | 143.70 | 0.40 | 4.90 | 108.30 | 0.40 | 7.46 | 13.20 | 16.40 | 7.89 | 1.40 | 0.88 | 34.64 | 25.30 | 0.47 |
| 0 | 5.31 | 11641.00 | 4.00 | 19.30 | 9.39 | 38.10 | 135.40 | 0.00 | 5.80 | 101.30 | 0.01 | 0.51 | 14.10 | 8.70 | 810.00 | 144.18 | 1.00 | 47.88 | 25.70 | 0.53 |
| 0 | 16.40 | 1351.20 | 155.50 | 12.90 | 13.34 | 16.50 | 121.90 | 0.10 | 3.90 | 89.30 | 0.57 | 11.61 | 13.70 | 21.60 | 15.50 | 3.88 | 0.00 | 490.57 | 26.00 | 0.00 |
| 0 | 3.93 | 161.60 | 0.50 | 24.56 | 9.39 | 47.42 | 139.30 | 11.10 | 8.78 | 105.40 | 0.98 | 6.26 | 13.60 | 25.96 | 2.60 | 0.65 | 0.50 | 2.73 | 27.54 | 0.00 |
| 0 | 1.12 | 208.99 | 0.00 | 44.00 | 6.24 | 33.70 | 134.10 | 5.60 | 7.70 | 101.80 | 0.38 | 6.71 | 13.70 | 53.90 | 433.79 | 77.21 | 6.15 | 127.08 | 23.40 | 0.78 |
| 0 | 5.31 | 2582.51 | 0.00 | 21.80 | 7.52 | 23.60 | 142.70 | 1.30 | 4.90 | 101.60 | 0.86 | 10.92 | 13.30 | 29.30 | 31.56 | 5.62 | 38.57 | 145.58 | 33.90 | 0.47 |
| 0 | 5.31 | 2948.00 | 1.00 | 11.90 | 5.88 | 19.60 | 144.30 | 2.80 | 5.90 | 106.00 | 0.50 | 8.52 | 12.30 | 14.20 | 3.00 | 0.53 | 0.00 | 33.82 | 25.10 | 0.18 |
| 1 | 1.87 | 2821.00 | 8.00 | 11.00 | 7.99 | 13.80 | 142.20 | 3.20 | 6.10 | 108.30 | 0.42 | 7.66 | 11.90 | 10.20 | 16.00 | 2.85 | 2.00 | 28.12 | 24.00 | 0.00 |
| 0 | 12.10 | 1910.00 | 1.00 | 15.80 | 12.45 | 97.00 | 135.70 | 0.60 | 4.70 | 101.90 | 0.71 | 8.19 | 12.40 | 13.20 | 102.00 | 18.16 | 64.00 | 29.19 | 28.20 | 0.18 |
| 1 | 0.34 | 1383.80 | 0.00 | 10.30 | 4.29 | 17.40 | 144.30 | 3.90 | 10.40 | 109.10 | 0.66 | 9.88 | 12.30 | 14.10 | 35.27 | 6.28 | 5.43 | 56.35 | 20.50 | 0.00 |
| 1 | 0.06 | 8000.00 | 0.00 | 15.50 | 21.15 | 15.30 | 136.30 | 0.30 | 5.40 | 98.20 | 0.27 | 6.75 | 12.40 | 17.50 | 0.00 | 0.00 | 80.00 | 1.75 | 26.80 | 0.00 |
| 1 | 0.48 | 578.00 | 0.00 | 10.10 | 4.63 | 22.50 | 144.80 | 8.30 | 4.70 | 107.60 | 0.42 | 7.85 | 13.70 | 11.00 | 16.00 | 2.85 | 22.00 | 1.96 | 21.00 | 0.18 |
| 1 | 0.56 | 8000.00 | 0.00 | 14.00 | 3.93 | 29.50 | 142.60 | 3.80 | 7.40 | 111.20 | 0.37 | 8.11 | 14.30 | 17.00 | 12.00 | 3.00 | 0.00 | 3.00 | 18.90 | 0.00 |
| 0 | 8.01 | 6436.43 | 1.10 | 14.80 | 6.73 | 44.70 | 131.20 | 1.10 | 20.30 | 94.20 | 0.36 | 3.35 | 13.20 | 32.00 | 15.43 | 2.75 | 578.62 | 30.60 | 48.60 | 0.00 |
| 0 | 21.30 | 6904.71 | 0.00 | 39.90 | 9.39 | 172.90 | 135.00 | 0.10 | 17.60 | 96.50 | 0.09 | 8.14 | 15.70 | 42.00 | 1.07 | 0.19 | 4.26 | 1.90 | 36.20 | 0.00 |
| 0 | 0.59 | 79.77 | 0.00 | 12.70 | 9.84 | 43.40 | 141.00 | 2.50 | 2.70 | 104.90 | 0.53 | 9.78 | 13.30 | 10.70 | 2.63 | 0.47 | 0.00 | 29.96 | 27.80 | 0.31 |
| 0 | 0.19 | 256.00 | 5.00 | 3.30 | 3.86 | 9.20 | 144.50 | 4.20 | 16.00 | 111.80 | 0.39 | 6.93 | 12.80 | 15.10 | 13.00 | 2.31 | 0.00 | 33.11 | 28.20 | 0.18 |
| 0 | 1.93 | 1040.54 | 0.00 | 29.50 | 4.51 | 31.40 | 143.70 | 2.30 | 7.50 | 109.20 | 0.34 | 5.79 | 12.70 | 24.50 | 44.71 | 7.96 | 1.75 | 16.38 | 30.10 | 0.47 |
| 0 | 5.31 | 237.00 | 0.00 | 8.10 | 8.29 | 15.20 | 143.50 | 3.00 | 6.20 | 106.00 | 0.41 | 5.73 | 13.20 | 9.00 | 13.20 | 3.30 | 164.50 | 5.75 | 22.60 | 0.60 |
| 0 | 30.56 | 994.96 | 0.00 | 17.60 | 10.56 | 213.80 | 129.30 | 0.00 | 15.50 | 96.80 | 0.77 | 27.93 | 12.50 | 39.50 | 386.59 | 68.81 | 20.16 | 305.83 | 21.70 | 0.16 |
| 1 | 2.10 | 3277.67 | 2.63 | 30.00 | 8.25 | 28.76 | 144.20 | 0.80 | 10.10 | 110.00 | 0.38 | 5.15 | 14.60 | 96.00 | 16.41 | 2.92 | 3.51 | 41.97 | 25.90 | 0.16 |
| 0 | 5.31 | 162.17 | 2.63 | 15.40 | 5.81 | 16.70 | 138.25 | 4.40 | 5.80 | 103.12 | 0.35 | 7.30 | 12.90 | 17.10 | 0.00 | 0.00 | 31.56 | 58.67 | 32.50 | 0.16 |
| 0 | 2.43 | 8000.00 | 0.00 | 43.00 | 12.84 | 68.30 | 135.20 | 0.60 | 5.20 | 102.60 | 0.66 | 9.59 | 14.30 | 29.10 | 0.00 | 0.00 | 0.00 | 78.00 | 23.70 | 0.00 |
| 1 | 1.87 | 511.06 | 0.00 | 12.30 | 8.37 | 16.10 | 140.60 | 1.90 | 5.70 | 105.80 | 0.30 | 6.80 | 12.20 | 13.20 | 71.13 | 12.66 | 1.76 | 83.47 | 32.60 | 0.00 |
| 0 | 5.31 | 1529.77 | 22.16 | 40.70 | 12.53 | 32.10 | 142.80 | 3.10 | 5.50 | 103.70 | 0.25 | 4.70 | 12.50 | 37.70 | 350.54 | 0.00 | 37.39 | 6.00 | 33.70 | 0.63 |
| 0 | 0.03 | 50.00 | 0.00 | 6.70 | 5.43 | 5.70 | 144.80 | 0.20 | 3.60 | 111.10 | 0.30 | 5.92 | 13.30 | 10.80 | 0.00 | 0.00 | 0.00 | 32.50 | 22.30 | 0.00 |
| 1 | 3.06 | 8000.00 | 0.00 | 13.50 | 5.81 | 47.50 | 135.70 | 0.20 | 16.10 | 106.10 | 0.35 | 4.01 | 13.30 | 16.30 | 0.00 | 0.00 | 0.00 | 2.75 | 20.90 | 0.00 |
| 1 | 0.53 | 28698.20 | 18.30 | 8.50 | 5.16 | 20.20 | 147.00 | 7.80 | 18.00 | 112.60 | 0.30 | 6.92 | 13.30 | 8.00 | 3.90 | 0.97 | 0.00 | 33.27 | 30.80 | 0.00 |
| 1 | 3.31 | 2545.44 | 6.18 | 9.30 | 7.64 | 33.50 | 138.10 | 0.70 | 16.90 | 100.60 | 1.04 | 13.32 | 14.00 | 8.60 | 71.59 | 12.82 | 15.12 | 60.16 | 28.90 | 0.23 |
| 0 | 0.27 | 565.42 | 18.41 | 12.00 | 5.68 | 19.20 | 139.40 | 0.60 | 4.80 | 103.10 | 0.45 | 6.42 | 12.90 | 12.80 | 16.66 | 2.97 | 2.63 | 66.94 | 20.90 | 0.16 |
| 0 | 5.31 | 1529.77 | 22.16 | 16.20 | 10.66 | 16.70 | 142.90 | 9.20 | 4.60 | 107.90 | 0.90 | 9.20 | 15.20 | 15.80 | 350.54 | 60.47 | 37.39 | 162.52 | 29.20 | 0.63 |
| 1 | 1.87 | 2545.44 | 6.18 | 8.00 | 4.48 | 14.90 | 138.50 | 1.50 | 9.70 | 103.00 | 0.28 | 7.09 | 15.20 | 13.50 | 71.59 | 12.82 | 15.12 | 60.16 | 37.30 | 0.23 |
| 1 | 0.10 | 1101.91 | 25.42 | 5.40 | 4.51 | 24.30 | 140.40 | 2.50 | 7.80 | 101.90 | 0.29 | 5.07 | 14.10 | 11.50 | 99.06 | 17.63 | 396.23 | 89.72 | 23.60 | 0.00 |
| 1 | 0.13 | 624.15 | 0.88 | 7.00 | 4.31 | 22.30 | 141.70 | 2.60 | 25.20 | 107.90 | 0.34 | 6.18 | 14.40 | 12.20 | 7.01 | 1.25 | 0.88 | 16.85 | 24.00 | 0.16 |
| 1 | 8.03 | 94.00 | 1.00 | 9.40 | 5.33 | 30.20 | 141.30 | 1.70 | 23.90 | 106.10 | 0.52 | 7.33 | 14.50 | 9.20 | 11.00 | 1.96 | 3.00 | 22.43 | 24.00 | 0.18 |
| 1 | 0.12 | 1113.45 | 0.00 | 6.60 | 8.62 | 12.90 | 142.30 | 0.50 | 10.10 | 110.40 | 0.35 | 5.13 | 13.40 | 10.20 | 3.51 | 0.62 | 2.63 | 2.34 | 17.20 | 0.00 |
| 0 | 0.93 | 5.27 | 34.25 | 15.00 | 5.08 | 47.42 | 144.10 | 1.60 | 14.20 | 108.80 | 0.32 | 7.60 | 13.30 | 12.00 | 84.30 | 15.01 | 0.00 | 435.62 | 23.40 | 1.09 |
| 1 | 8.98 | 995.00 | 0.00 | 5.30 | 9.07 | 18.70 | 130.60 | 1.20 | 3.20 | 98.50 | 0.69 | 9.85 | 21.00 | 5.90 | 8.00 | 1.42 | 0.00 | 13.71 | 24.90 | 0.18 |
| 0 | 0.17 | 509.00 | 21.00 | 15.90 | 9.16 | 47.30 | 141.10 | 0.80 | 20.10 | 102.70 | 0.75 | 10.15 | 13.80 | 14.00 | 135.00 | 24.03 | 2.00 | 120.51 | 25.20 | 0.53 |
| 1 | 1.19 | 4.38 | 0.00 | 13.80 | 5.97 | 20.80 | 141.00 | 1.00 | 9.70 | 103.70 | 0.57 | 5.77 | 14.70 | 17.80 | 1.75 | 0.31 | 0.00 | 1.72 | 34.20 | 0.00 |
| 0 | 0.52 | 3.51 | 0.00 | 34.00 | 10.20 | 47.42 | 144.00 | 2.30 | 7.50 | 106.50 | 0.31 | 5.40 | 13.00 | 11.00 | 7.90 | 1.41 | 0.88 | 6.25 | 34.00 | 2.34 |
| 1 | 1.87 | 61.36 | 3.51 | 21.60 | 7.64 | 28.70 | 135.30 | 0.90 | 11.40 | 103.30 | 0.43 | 6.05 | 12.90 | 24.30 | 13.15 | 2.34 | 1.75 | 9.52 | 40.30 | 0.31 |
| 1 | 3.91 | 573.41 | 0.00 | 10.40 | 7.64 | 113.00 | 142.30 | 0.00 | 15.90 | 103.20 | 0.45 | 6.30 | 13.90 | 14.50 | 6.15 | 1.09 | 0.88 | 5.00 | 32.60 | 0.00 |
| 1 | 9.30 | 6209.00 | 2.00 | 40.90 | 10.54 | 57.00 | 139.10 | 3.00 | 5.60 | 100.40 | 0.50 | 9.45 | 12.70 | 35.00 | 32.00 | 5.70 | 18.00 | 21.18 | 35.00 | 0.18 |
| 0 | 1.17 | 8000.00 | 0.00 | 34.60 | 7.53 | 47.60 | 144.60 | 2.30 | 4.80 | 108.40 | 0.30 | 5.50 | 12.70 | 28.60 | 0.00 | 0.00 | 0.00 | 13.75 | 22.10 | 0.00 |
| 1 | 1.30 | 2545.44 | 6.18 | 10.00 | 4.47 | 25.00 | 142.00 | 0.30 | 5.90 | 103.80 | 0.40 | 7.41 | 11.80 | 11.10 | 71.59 | 12.82 | 15.12 | 60.16 | 28.00 | 0.23 |
| 0 | 0.28 | 118.34 | 0.00 | 33.40 | 6.32 | 22.20 | 143.40 | 0.50 | 6.90 | 113.50 | 0.54 | 9.36 | 13.50 | 27.20 | 7.01 | 1.25 | 0.00 | 14.04 | 25.40 | 0.00 |
| 0 | 0.24 | 179.71 | 0.00 | 29.20 | 10.16 | 21.50 | 141.60 | 0.40 | 6.60 | 111.50 | 0.54 | 7.90 | 13.00 | 22.20 | 13.15 | 2.34 | 0.00 | 24.50 | 23.60 | 0.00 |
| 1 | 1.87 | 69.10 | 3.90 | 7.20 | 3.50 | 21.20 | 138.60 | 4.40 | 9.80 | 102.80 | 0.38 | 5.96 | 12.50 | 12.20 | 1.70 | 0.42 | 1.00 | 17.30 | 22.00 | 0.13 |
| 0 | 0.46 | 799.09 | 0.00 | 24.56 | 5.03 | 47.42 | 131.70 | 0.40 | 8.78 | 95.10 | 0.68 | 9.57 | 12.10 | 25.96 | 8.78 | 1.56 | 36.00 | 15.16 | 27.54 | 0.16 |
| 0 | 1.13 | 8000.00 | 31.00 | 14.00 | 4.72 | 72.30 | 133.40 | 0.40 | 15.70 | 101.10 | 0.49 | 7.80 | 16.40 | 24.90 | 0.00 | 0.00 | 0.00 | 450.00 | 31.00 | 0.00 |
| 1 | 2.95 | 2785.60 | 5.50 | 9.40 | 4.34 | 116.30 | 141.00 | 2.40 | 9.10 | 107.10 | 0.32 | 5.98 | 14.40 | 17.20 | 133.70 | 33.42 | 0.50 | 46.25 | 36.90 | 0.00 |
| 0 | 19.20 | 185.50 | 1.00 | 10.80 | 5.60 | 28.40 | 137.40 | 0.20 | 22.30 | 115.70 | 0.08 | 5.35 | 13.30 | 17.80 | 9.20 | 2.30 | 0.50 | 18.48 | 24.00 | 0.38 |
| 0 | 1.19 | 1529.77 | 22.16 | 10.30 | 9.39 | 232.10 | 132.60 | 3.40 | 13.60 | 105.90 | 0.72 | 11.63 | 15.40 | 18.30 | 350.54 | 60.47 | 37.39 | 162.52 | 34.70 | 0.63 |
| 1 | 1.87 | 2545.44 | 6.18 | 6.50 | 7.64 | 19.10 | 135.70 | 4.60 | 7.30 | 98.30 | 0.30 | 6.68 | 17.70 | 9.60 | 71.59 | 12.82 | 15.12 | 60.16 | 20.80 | 0.23 |
| 1 | 1.63 | 2545.44 | 6.18 | 7.60 | 10.88 | 23.60 | 129.50 | 17.00 | 5.90 | 93.60 | 0.12 | 9.74 | 19.90 | 8.70 | 71.59 | 12.82 | 15.12 | 60.16 | 21.60 | 0.23 |
| 1 | 1.69 | 8000.00 | 0.00 | 16.00 | 3.52 | 42.90 | 136.50 | 4.20 | 21.40 | 101.60 | 0.48 | 3.78 | 17.70 | 14.40 | 0.00 | 0.00 | 0.00 | 8.25 | 29.90 | 0.00 |
| 1 | 0.12 | 949.38 | 1.75 | 11.80 | 5.58 | 9.10 | 143.70 | 0.60 | 3.30 | 108.80 | 0.23 | 2.44 | 13.20 | 18.20 | 129.74 | 23.09 | 30.68 | 28.55 | 19.40 | 0.16 |
| 1 | 6.27 | 5576.00 | 1.00 | 7.70 | 9.67 | 19.10 | 122.60 | 1.20 | 17.40 | 91.10 | 0.39 | 6.86 | 13.30 | 13.90 | 485.00 | 86.33 | 1.00 | 116.23 | 37.80 | 0.89 |
| 1 | 0.37 | 8000.00 | 0.00 | 18.70 | 6.38 | 33.70 | 142.40 | 0.70 | 6.20 | 108.90 | 0.27 | 8.44 | 13.00 | 14.10 | 0.00 | 0.00 | 5.00 | 0.00 | 26.50 | 0.00 |
| 1 | 3.74 | 254.95 | 0.00 | 9.60 | 6.62 | 51.10 | 127.60 | 0.70 | 4.40 | 91.80 | 0.67 | 9.55 | 12.50 | 10.40 | 24.62 | 4.38 | 9.67 | 56.02 | 25.60 | 0.47 |
| 0 | 11.60 | 802.00 | 0.00 | 15.20 | 13.06 | 16.60 | 135.50 | 1.10 | 5.20 | 96.00 | 0.28 | 5.92 | 13.00 | 15.70 | 7.00 | 1.25 | 1.00 | 16.02 | 23.50 | 0.18 |
| 1 | 0.45 | 2052.16 | 0.00 | 50.50 | 7.84 | 58.90 | 144.10 | 1.80 | 6.40 | 104.90 | 0.56 | 9.44 | 13.40 | 47.70 | 189.35 | 33.70 | 34.19 | 20.13 | 29.90 | 0.16 |
| 0 | 2.12 | 2553.80 | 1362.90 | 8.00 | 6.03 | 12.27 | 137.30 | 0.10 | 27.90 | 101.10 | 0.36 | 11.73 | 13.40 | 12.90 | 11.60 | 2.90 | 0.00 | 5222.88 | 25.40 | 0.80 |
| 1 | 0.17 | 0.00 | 0.00 | 17.90 | 8.67 | 18.00 | 143.40 | 0.90 | 4.60 | 105.90 | 0.48 | 5.89 | 12.60 | 14.20 | 8.77 | 1.56 | 45.58 | 11.86 | 24.00 | 0.62 |
| 0 | 0.46 | 2146.90 | 168.80 | 8.70 | 6.17 | 12.20 | 141.10 | 3.80 | 6.90 | 111.50 | 0.26 | 7.94 | 13.20 | 11.50 | 13.00 | 3.25 | 0.00 | 408.95 | 20.90 | 1.98 |
| 1 | 0.33 | 504.00 | 9.00 | 11.60 | 8.47 | 30.10 | 141.10 | 2.40 | 8.80 | 109.00 | 0.42 | 7.36 | 13.50 | 13.80 | 40.00 | 7.12 | 1.00 | 44.50 | 34.30 | 0.00 |
| 1 | 4.38 | 8000.00 | 0.00 | 10.40 | 6.09 | 17.60 | 140.30 | 1.00 | 7.20 | 105.20 | 0.36 | 7.39 | 14.50 | 17.40 | 0.00 | 0.00 | 5.00 | 40.00 | 25.50 | 0.00 |
| 0 | 0.40 | 2583.00 | 20.00 | 58.10 | 7.30 | 21.00 | 144.60 | 3.40 | 6.80 | 113.20 | 0.32 | 7.26 | 16.20 | 39.20 | 72.00 | 12.82 | 0.00 | 45.03 | 33.40 | 0.36 |
| 1 | 1.87 | 2545.44 | 6.18 | 14.20 | 10.70 | 12.30 | 142.10 | 1.90 | 4.50 | 103.00 | 0.15 | 5.20 | 12.40 | 11.50 | 71.59 | 0.00 | 15.12 | 56.00 | 22.50 | 0.23 |
| 1 | 0.17 | 2507.00 | 17.00 | 18.00 | 19.09 | 14.10 | 139.20 | 1.50 | 6.30 | 103.60 | 0.14 | 4.73 | 12.60 | 13.90 | 25.00 | 4.45 | 1.00 | 34.00 | 28.10 | 0.00 |
| 1 | 0.45 | 2230.17 | 22.94 | 8.50 | 4.88 | 20.40 | 139.60 | 1.20 | 28.10 | 105.60 | 0.48 | 6.70 | 13.10 | 20.60 | 38.08 | 6.78 | 218.78 | 416.91 | 25.10 | 0.00 |
| 0 | 1.09 | 25.42 | 1.75 | 22.20 | 7.46 | 37.80 | 137.60 | 1.50 | 4.00 | 101.40 | 0.22 | 5.98 | 12.80 | 22.50 | 5.26 | 0.94 | 0.00 | 9.05 | 26.50 | 0.00 |
| 1 | 0.51 | 60.90 | 0.00 | 10.50 | 12.43 | 20.40 | 143.10 | 2.20 | 6.70 | 107.30 | 0.35 | 6.66 | 12.40 | 9.20 | 0.00 | 0.00 | 2.40 | 1.88 | 28.60 | 0.25 |
| 0 | 2.26 | 82.32 | 25.66 | 18.60 | 9.39 | 47.42 | 134.40 | 0.00 | 23.30 | 85.50 | 0.53 | 14.37 | 14.40 | 54.30 | 629.67 | 112.08 | 0.00 | 883.32 | 25.00 | 0.19 |
| 0 | 5.23 | 67.76 | 58.52 | 39.20 | 11.15 | 96.40 | 136.00 | 1.30 | 21.10 | 100.70 | 0.59 | 11.47 | 14.20 | 39.00 | 23528.14 | 4188.01 | 6.16 | 646.92 | 22.90 | 1.10 |
| 0 | 5.31 | 812.90 | 11.70 | 9.90 | 4.44 | 74.80 | 132.70 | 0.30 | 15.40 | 100.10 | 0.89 | 9.51 | 14.60 | 9.20 | 5.20 | 1.30 | 0.00 | 91.40 | 30.20 | 0.25 |
| 0 | 2.99 | 17003.00 | 25.00 | 5.50 | 11.85 | 19.60 | 138.10 | 0.10 | 4.40 | 104.90 | 0.17 | 4.75 | 14.00 | 9.20 | 719.00 | 127.98 | 27.00 | 70.31 | 26.60 | 0.18 |
| 1 | 4.11 | 2545.44 | 6.18 | 11.80 | 6.67 | 31.80 | 145.30 | 5.40 | 18.80 | 119.70 | 0.26 | 3.24 | 13.00 | 10.00 | 71.59 | 12.82 | 15.12 | 60.16 | 25.20 | 0.23 |
| 0 | 1.06 | 0.00 | 0.00 | 12.40 | 8.32 | 168.80 | 143.30 | 1.20 | 3.50 | 108.50 | 0.15 | 2.24 | 13.40 | 21.80 | 0.00 | 0.00 | 0.00 | 1.25 | 28.90 | 0.00 |
| 0 | 0.27 | 3.51 | 8.77 | 11.00 | 6.43 | 47.42 | 145.50 | 2.20 | 5.60 | 104.30 | 0.50 | 5.31 | 12.90 | 17.00 | 71.01 | 12.64 | 85.03 | 138.25 | 26.70 | 0.62 |
| 1 | 2.48 | 584.00 | 0.00 | 17.60 | 4.95 | 28.40 | 144.90 | 4.30 | 8.50 | 109.80 | 0.46 | 7.82 | 13.00 | 16.70 | 17.00 | 3.03 | 1.00 | 21.00 | 19.10 | 0.00 |
| 1 | 0.30 | 14.90 | 0.00 | 16.40 | 5.46 | 97.80 | 144.90 | 3.30 | 17.40 | 116.10 | 0.39 | 4.84 | 12.80 | 19.20 | 8.77 | 1.56 | 7.89 | 1.56 | 18.50 | 0.00 |
| 1 | 1.87 | 8000.00 | 33.00 | 5.20 | 7.92 | 9.60 | 141.30 | 0.20 | 9.70 | 113.10 | 0.24 | 7.38 | 14.20 | 10.20 | 0.00 | 0.00 | 0.00 | 127.75 | 25.10 | 0.00 |
| 1 | 4.43 | 1776.00 | 0.00 | 7.60 | 3.92 | 19.90 | 138.80 | 0.40 | 42.40 | 99.90 | 0.49 | 8.44 | 14.20 | 12.30 | 1345.00 | 239.41 | 3.00 | 23.50 | 23.10 | 0.18 |
| 1 | 1.42 | 13357.28 | 2.06 | 31.10 | 10.29 | 34.10 | 136.80 | 1.80 | 11.90 | 106.90 | 0.26 | 9.77 | 12.50 | 22.40 | 33.03 | 5.88 | 6.19 | 51.81 | 36.70 | 0.00 |
| 1 | 0.76 | 3228.58 | 1.75 | 30.70 | 6.52 | 60.50 | 140.10 | 0.10 | 4.40 | 102.40 | 0.49 | 10.79 | 13.00 | 18.40 | 6.14 | 1.09 | 0.88 | 9.99 | 30.90 | 0.31 |
| 0 | 0.37 | 2202.06 | 7.01 | 17.00 | 3.59 | 18.00 | 146.00 | 5.10 | 19.80 | 106.50 | 1.02 | 11.82 | 13.00 | 18.60 | 35.06 | 6.24 | 1.75 | 57.11 | 28.90 | 0.31 |
| 0 | 0.33 | 64.87 | 0.00 | 57.20 | 6.21 | 13.20 | 140.60 | 4.30 | 4.40 | 105.20 | 0.34 | 4.12 | 12.40 | 106.10 | 10.52 | 1.87 | 8.77 | 5.77 | 26.90 | 0.00 |
| 1 | 0.19 | 337.50 | 3.51 | 3.00 | 6.27 | 13.60 | 141.00 | 8.10 | 5.40 | 104.60 | 0.25 | 3.82 | 12.60 | 17.20 | 11.40 | 2.03 | 7.89 | 14.67 | 30.60 | 0.00 |
| 0 | 5.31 | 8000.00 | 0.00 | 63.20 | 8.84 | 58.20 | 141.00 | 0.80 | 9.20 | 106.50 | 0.90 | 10.80 | 13.50 | 21.30 | 0.00 | 0.00 | 0.00 | 50.75 | 26.90 | 0.00 |
| 0 | 5.12 | 7.01 | 0.00 | 7.60 | 19.31 | 39.60 | 135.40 | 2.80 | 23.70 | 103.80 | 0.41 | 6.64 | 12.80 | 10.90 | 163.05 | 29.02 | 1.75 | 553.78 | 24.90 | 0.78 |
| 1 | 0.30 | 28.05 | 0.00 | 9.70 | 7.48 | 15.80 | 144.20 | 1.00 | 4.80 | 109.90 | 0.39 | 6.83 | 12.80 | 11.00 | 9.64 | 1.72 | 2.63 | 149.02 | 22.40 | 0.31 |
| 0 | 0.50 | 0.00 | 0.00 | 16.00 | 13.63 | 50.20 | 138.20 | 1.50 | 4.80 | 104.70 | 0.36 | 6.74 | 12.70 | 16.50 | 5.26 | 0.94 | 0.00 | 3.12 | 30.40 | 0.00 |
| 0 | 0.14 | 110.45 | 0.00 | 9.10 | 9.75 | 25.30 | 143.00 | 1.20 | 9.90 | 109.10 | 0.44 | 6.79 | 13.10 | 17.30 | 95.55 | 17.01 | 24.55 | 21.85 | 30.20 | 0.00 |
| 1 | 3.89 | 817.01 | 0.00 | 11.30 | 4.25 | 29.10 | 136.00 | 1.70 | 8.80 | 105.40 | 0.08 | 6.25 | 16.20 | 15.20 | 127.11 | 22.63 | 9.64 | 207.06 | 34.90 | 0.47 |
| 1 | 0.37 | 280.00 | 0.00 | 20.90 | 5.32 | 18.80 | 142.10 | 0.20 | 10.00 | 104.00 | 0.42 | 5.46 | 15.20 | 16.80 | 5.00 | 0.89 | 0.00 | 1.60 | 19.40 | 0.36 |
| 1 | 6.38 | 0.00 | 0.00 | 7.00 | 5.98 | 13.70 | 141.30 | 0.10 | 22.00 | 115.40 | 0.45 | 6.95 | 13.50 | 7.70 | 0.00 | 0.00 | 0.00 | 0.00 | 23.10 | 0.00 |
| 0 | 2.05 | 1492.00 | 0.00 | 8.60 | 28.88 | 21.70 | 134.80 | 1.90 | 12.10 | 101.50 | 0.27 | 5.21 | 13.30 | 13.50 | 3.00 | 0.53 | 0.00 | 3.38 | 26.00 | 0.36 |
| 0 | 5.31 | 1529.77 | 22.16 | 24.56 | 8.10 | 47.42 | 135.60 | 2.40 | 8.78 | 96.20 | 0.26 | 4.80 | 12.20 | 15.00 | 350.54 | 0.00 | 37.39 | 5.00 | 27.54 | 0.63 |
| 1 | 0.27 | 14.03 | 0.00 | 9.50 | 6.28 | 20.50 | 142.20 | 1.70 | 13.50 | 111.80 | 0.02 | 5.88 | 13.00 | 11.50 | 4.38 | 0.78 | 0.88 | 3.28 | 21.10 | 0.16 |
| 0 | 0.33 | 0.00 | 0.00 | 22.80 | 9.59 | 34.40 | 136.60 | 0.80 | 3.80 | 97.30 | 0.32 | 5.46 | 12.10 | 20.50 | 3.52 | 0.63 | 3.52 | 14.08 | 22.80 | 0.00 |
| 1 | 0.70 | 380.00 | 2.00 | 6.80 | 5.14 | 12.70 | 142.30 | 1.70 | 13.00 | 112.00 | 0.19 | 5.08 | 15.70 | 14.70 | 8.00 | 1.42 | 0.00 | 11.21 | 38.40 | 0.00 |
| 1 | 0.18 | 82.40 | 0.00 | 81.00 | 8.93 | 42.60 | 140.60 | 2.30 | 3.10 | 103.90 | 0.55 | 6.92 | 12.80 | 45.70 | 12.43 | 2.21 | 33.31 | 5.46 | 26.60 | 0.00 |
| 0 | 46.30 | 97.00 | 0.00 | 95.30 | 15.19 | 32.40 | 134.70 | 0.00 | 46.50 | 90.90 | 0.87 | 40.63 | 14.90 | 374.60 | 15268.00 | 2717.70 | 13.00 | 200.43 | 22.70 | 1.07 |
| 1 | 1.87 | 0.00 | 0.00 | 8.90 | 5.94 | 18.10 | 140.80 | 2.80 | 7.50 | 105.80 | 0.38 | 5.70 | 12.20 | 13.10 | 20.00 | 5.00 | 0.00 | 79.25 | 23.90 | 0.00 |
| 1 | 0.05 | 106.00 | 9.00 | 18.00 | 15.20 | 31.40 | 140.90 | 2.00 | 6.30 | 103.80 | 0.40 | 6.29 | 17.90 | 23.00 | 83.00 | 14.77 | 2.00 | 51.44 | 36.70 | 0.53 |
| 0 | 0.26 | 1469.70 | 464.20 | 33.90 | 9.86 | 43.60 | 142.00 | 0.90 | 6.20 | 106.60 | 0.37 | 7.77 | 17.10 | 36.30 | 6.80 | 1.70 | 0.00 | 1309.45 | 30.80 | 1.05 |
| 0 | 15.90 | 44.00 | 0.00 | 6.60 | 16.15 | 29.70 | 135.70 | 0.00 | 9.80 | 103.50 | 0.08 | 9.01 | 12.80 | 12.40 | 76.00 | 13.53 | 7.00 | 42.19 | 24.20 | 0.00 |
| 1 | 1.23 | 1.00 | 0.00 | 29.00 | 8.53 | 48.90 | 143.00 | 1.00 | 9.50 | 107.40 | 0.23 | 3.80 | 12.90 | 33.10 | 13.00 | 2.31 | 0.00 | 2.31 | 32.00 | 1.07 |
| 1 | 2.85 | 90.29 | 0.00 | 8.10 | 5.25 | 17.70 | 137.20 | 1.10 | 4.10 | 101.40 | 0.34 | 7.72 | 17.40 | 20.40 | 18.41 | 3.28 | 1.75 | 61.32 | 34.60 | 0.00 |
| 0 | 1.06 | 22.79 | 8.77 | 15.60 | 8.90 | 19.80 | 145.90 | 0.80 | 3.60 | 108.70 | 0.37 | 7.62 | 13.30 | 16.80 | 83.28 | 14.82 | 14.03 | 138.41 | 23.70 | 0.16 |
| 0 | 4.37 | 618.00 | 53.00 | 5.60 | 7.14 | 17.10 | 141.20 | 0.10 | 12.90 | 107.10 | 0.44 | 9.64 | 13.70 | 8.30 | 61.00 | 10.86 | 1.00 | 96.48 | 26.90 | 0.00 |
| 0 | 18.54 | 147.50 | 23.90 | 18.70 | 7.92 | 39.90 | 138.40 | 0.30 | 8.60 | 104.50 | 0.53 | 8.73 | 13.20 | 39.00 | 295.10 | 73.78 | 6.30 | 73.20 | 17.50 | 0.38 |
| 1 | 0.24 | 8000.00 | 0.00 | 20.20 | 7.08 | 31.80 | 143.90 | 3.10 | 6.90 | 108.70 | 0.21 | 4.98 | 13.50 | 22.90 | 0.00 | 0.00 | 20.00 | 9.75 | 19.80 | 0.00 |
| 1 | 0.36 | 50.00 | 0.00 | 30.30 | 6.45 | 36.10 | 145.50 | 5.00 | 5.10 | 114.60 | 0.16 | 2.51 | 13.90 | 29.50 | 0.00 | 0.00 | 20.00 | 102.00 | 16.90 | 0.00 |
| 0 | 0.52 | 774.93 | 6.14 | 11.60 | 4.26 | 20.30 | 145.00 | 1.70 | 18.90 | 105.10 | 0.45 | 8.00 | 12.50 | 16.20 | 9.64 | 1.72 | 1.75 | 35.73 | 24.80 | 0.31 |
| 1 | 1.87 | 3422.00 | 0.00 | 11.40 | 5.44 | 34.00 | 140.00 | 7.00 | 8.90 | 105.60 | 0.34 | 4.53 | 13.50 | 13.80 | 12.00 | 2.14 | 3.00 | 6.23 | 23.90 | 0.00 |
| 0 | 6.01 | 1529.77 | 22.16 | 7.70 | 12.26 | 150.80 | 137.50 | 0.40 | 37.10 | 102.00 | 0.82 | 10.01 | 15.80 | 9.20 | 350.54 | 60.47 | 37.39 | 162.52 | 22.30 | 0.63 |
| 1 | 1.87 | 776.26 | 0.88 | 14.00 | 7.64 | 28.76 | 143.50 | 3.00 | 5.80 | 102.50 | 0.42 | 6.89 | 12.30 | 20.00 | 21.98 | 3.91 | 1.76 | 19.87 | 33.40 | 0.16 |
| 1 | 0.10 | 310.32 | 6.14 | 14.50 | 5.32 | 24.50 | 143.50 | 3.30 | 10.10 | 108.40 | 0.29 | 6.74 | 12.70 | 12.30 | 4.38 | 0.78 | 3.51 | 23.09 | 23.70 | 0.00 |
| 1 | 4.68 | 1061.57 | 0.00 | 23.10 | 10.44 | 25.80 | 139.90 | 0.70 | 26.10 | 103.90 | 0.43 | 4.25 | 13.40 | 30.30 | 239.21 | 42.58 | 0.00 | 71.64 | 32.30 | 0.00 |
| 1 | 0.45 | 742.00 | 0.00 | 16.70 | 9.09 | 29.50 | 147.70 | 3.20 | 12.70 | 108.40 | 0.38 | 7.15 | 14.10 | 16.20 | 9.00 | 1.60 | 0.00 | 2.85 | 34.30 | 0.00 |
| 0 | 1.16 | 31.00 | 2.00 | 19.30 | 8.99 | 23.60 | 144.20 | 2.40 | 3.80 | 105.40 | 0.50 | 8.85 | 13.00 | 13.00 | 12.00 | 2.14 | 0.00 | 21.54 | 26.40 | 0.00 |
| 0 | 0.36 | 12.18 | 0.00 | 13.00 | 10.51 | 12.90 | 144.60 | 2.00 | 12.10 | 110.30 | 0.27 | 5.72 | 13.20 | 17.90 | 6.96 | 1.24 | 0.87 | 4.18 | 31.40 | 0.00 |
| 0 | 0.32 | 1529.77 | 22.16 | 9.40 | 5.15 | 19.90 | 138.60 | 0.30 | 9.70 | 104.20 | 0.29 | 7.40 | 12.80 | 28.90 | 350.54 | 0.00 | 37.39 | 7.00 | 23.50 | 0.63 |
| 0 | 4.92 | 50.00 | 0.00 | 10.10 | 15.51 | 22.20 | 137.10 | 1.40 | 5.40 | 103.40 | 0.80 | 10.85 | 13.20 | 15.50 | 0.00 | 0.00 | 5.00 | 86.25 | 27.90 | 0.00 |
| 0 | 0.40 | 143.77 | 21.92 | 23.80 | 17.88 | 55.30 | 138.60 | 5.50 | 3.90 | 103.80 | 0.36 | 5.66 | 12.70 | 21.10 | 469.87 | 83.64 | 1.75 | 681.57 | 26.40 | 0.16 |
| 0 | 44.10 | 1529.77 | 22.16 | 12.80 | 9.39 | 134.00 | 129.30 | 0.00 | 7.90 | 100.20 | 0.00 | 0.22 | 17.40 | 11.10 | 350.54 | 60.47 | 37.39 | 162.52 | 22.00 | 0.63 |
| 1 | 4.64 | 1.75 | 0.00 | 8.30 | 4.51 | 18.00 | 129.60 | 1.00 | 7.00 | 97.10 | 0.39 | 7.12 | 17.30 | 15.90 | 98.18 | 17.48 | 0.88 | 8.43 | 18.80 | 0.31 |
| 1 | 1.87 | 0.00 | 0.00 | 21.70 | 8.56 | 28.30 | 139.90 | 1.40 | 4.70 | 105.10 | 0.55 | 5.28 | 13.00 | 14.50 | 8.00 | 1.42 | 20.00 | 0.53 | 29.60 | 0.00 |
| 0 | 1.21 | 13.15 | 0.00 | 7.50 | 6.94 | 50.50 | 140.70 | 1.60 | 5.00 | 100.50 | 0.48 | 5.00 | 13.30 | 11.60 | 12.27 | 2.18 | 0.88 | 3.28 | 30.80 | 0.00 |
| 1 | 9.15 | 4210.40 | 0.00 | 440.70 | 3.78 | 56.50 | 126.20 | 0.40 | 16.90 | 88.00 | 0.23 | 11.88 | 18.90 | 1386.50 | 100.48 | 17.89 | 7.35 | 250.83 | 31.50 | 8.07 |
| 0 | 9.50 | 0.00 | 0.00 | 96.70 | 10.05 | 53.30 | 138.00 | 0.10 | 12.10 | 105.10 | 0.89 | 11.11 | 13.20 | 24.00 | 35.00 | 8.75 | 5.00 | 11.00 | 41.00 | 0.00 |
| 0 | 9.38 | 0.87 | 0.00 | 21.70 | 15.20 | 43.40 | 137.80 | 0.10 | 5.10 | 104.30 | 0.64 | 9.58 | 13.20 | 16.00 | 20.00 | 3.56 | 1.74 | 2.32 | 28.30 | 0.15 |
| 1 | 7.68 | 7.01 | 11.40 | 8.10 | 5.59 | 14.00 | 140.00 | 1.20 | 2.90 | 101.20 | 0.34 | 6.45 | 14.10 | 15.60 | 15.78 | 2.81 | 10.52 | 36.36 | 28.30 | 0.31 |
| 0 | 0.44 | 599.70 | 8.30 | 29.90 | 9.04 | 23.20 | 139.50 | 1.90 | 5.30 | 103.20 | 0.57 | 8.88 | 14.40 | 26.70 | 4.50 | 1.13 | 6.80 | 58.83 | 40.00 | 1.70 |
| 1 | 1.87 | 2545.44 | 6.18 | 10.30 | 5.62 | 17.90 | 144.90 | 0.80 | 5.50 | 104.80 | 0.47 | 9.20 | 12.60 | 22.40 | 71.59 | 0.00 | 15.12 | 0.00 | 25.90 | 0.23 |
| 0 | 0.17 | 4273.80 | 66.90 | 14.20 | 8.23 | 18.40 | 139.40 | 1.00 | 7.80 | 102.80 | 0.50 | 6.69 | 12.80 | 19.00 | 18.80 | 4.70 | 109.50 | 1421.58 | 29.10 | 2.42 |
| 1 | 0.63 | 2676.70 | 0.00 | 14.50 | 5.62 | 20.40 | 141.60 | 2.10 | 6.70 | 104.10 | 0.46 | 6.30 | 12.20 | 18.40 | 31.40 | 7.85 | 66.20 | 3.50 | 23.70 | 0.13 |
| 1 | 0.38 | 7.01 | 0.00 | 6.20 | 13.18 | 13.20 | 144.50 | 2.00 | 15.20 | 107.90 | 0.34 | 7.21 | 13.50 | 8.60 | 7.89 | 1.40 | 0.88 | 0.47 | 19.80 | 0.00 |
| 1 | 1.87 | 2545.44 | 6.18 | 17.30 | 5.90 | 23.70 | 141.50 | 1.30 | 6.50 | 104.00 | 0.30 | 5.92 | 12.20 | 14.20 | 71.59 | 12.82 | 15.12 | 60.16 | 28.70 | 0.23 |
| 1 | 0.54 | 2545.44 | 6.18 | 12.70 | 7.79 | 18.10 | 129.90 | 0.30 | 5.00 | 96.90 | 0.14 | 6.90 | 22.40 | 11.30 | 71.59 | 12.82 | 15.12 | 60.16 | 21.20 | 0.23 |
| 1 | 1.87 | 2545.44 | 6.18 | 39.30 | 7.48 | 27.10 | 142.60 | 7.00 | 4.00 | 103.50 | 0.62 | 7.16 | 12.40 | 23.10 | 71.59 | 12.82 | 15.12 | 60.16 | 26.20 | 0.23 |
| 0 | 0.05 | 8000.00 | 0.00 | 12.50 | 8.51 | 16.40 | 138.30 | 0.50 | 7.20 | 103.80 | 0.27 | 4.18 | 13.20 | 15.20 | 6.00 | 1.50 | 5.00 | 31.50 | 19.30 | 0.00 |
| 0 | 12.10 | 0.00 | 0.00 | 6.00 | 6.63 | 30.00 | 136.30 | 0.20 | 10.50 | 104.50 | 0.39 | 9.53 | 14.80 | 15.00 | 2.00 | 0.50 | 0.00 | 2.75 | 34.60 | 0.00 |
| 0 | 0.20 | 271.34 | 4.39 | 12.30 | 6.29 | 14.00 | 142.80 | 1.60 | 9.40 | 108.40 | 0.64 | 9.22 | 13.30 | 15.60 | 28.10 | 5.00 | 0.00 | 16.57 | 29.60 | 0.00 |
| 0 | 0.25 | 0.88 | 0.00 | 9.90 | 5.66 | 32.90 | 142.70 | 1.10 | 5.60 | 109.30 | 0.42 | 5.16 | 12.70 | 16.20 | 12.27 | 2.18 | 0.00 | 7.49 | 25.70 | 0.00 |
| 1 | 1.59 | 170.00 | 8.00 | 14.40 | 4.07 | 26.70 | 144.70 | 0.20 | 7.50 | 104.00 | 0.64 | 8.44 | 12.80 | 21.40 | 22.00 | 3.92 | 0.00 | 61.05 | 31.80 | 0.00 |
| 0 | 1.59 | 1529.77 | 22.16 | 47.00 | 14.33 | 80.00 | 142.80 | 0.00 | 6.00 | 104.40 | 0.35 | 6.93 | 12.70 | 29.00 | 350.54 | 60.47 | 37.39 | 162.52 | 28.80 | 0.63 |
| 0 | 1.80 | 112.21 | 0.00 | 10.30 | 5.57 | 33.20 | 136.50 | 0.40 | 4.80 | 102.90 | 0.28 | 5.06 | 17.40 | 21.70 | 371.69 | 66.16 | 243.70 | 843.07 | 22.10 | 0.00 |
| 1 | 0.29 | 14299.10 | 15.60 | 7.70 | 4.32 | 14.70 | 142.90 | 2.50 | 5.00 | 108.80 | 0.25 | 5.05 | 12.30 | 14.10 | 2.10 | 0.53 | 0.00 | 56.40 | 19.50 | 0.47 |
| 1 | 9.50 | 8000.00 | 0.00 | 12.10 | 7.64 | 87.00 | 133.90 | 3.90 | 7.20 | 102.70 | 0.47 | 5.14 | 18.40 | 21.60 | 81.00 | 20.25 | 20.00 | 26.00 | 23.40 | 0.00 |
| 0 | 19.34 | 331.36 | 3.51 | 20.40 | 8.10 | 46.10 | 141.60 | 1.40 | 8.20 | 109.70 | 0.44 | 10.25 | 15.80 | 17.10 | 16.66 | 2.97 | 0.00 | 82.23 | 27.70 | 0.00 |
| 1 | 0.64 | 5993.84 | 0.00 | 11.80 | 7.77 | 21.80 | 143.00 | 2.60 | 5.50 | 106.90 | 0.42 | 8.85 | 13.60 | 12.70 | 44.89 | 7.99 | 11.51 | 85.24 | 29.10 | 0.00 |
| 1 | 1.09 | 16.66 | 0.00 | 20.30 | 8.87 | 31.30 | 139.70 | 4.20 | 4.00 | 101.30 | 0.32 | 6.13 | 12.20 | 18.60 | 35.06 | 6.24 | 129.74 | 1.40 | 22.20 | 0.16 |
| 1 | 0.76 | 103.44 | 0.00 | 11.40 | 5.98 | 24.70 | 143.60 | 2.25 | 4.90 | 112.70 | 0.40 | 6.85 | 14.01 | 16.10 | 9.64 | 1.72 | 49.09 | 0.78 | 19.20 | 0.00 |
