# Supplementary material for: A personalized prediction model for distinguishing between asymptomatic bacteriuria and symptomatic urinary tract infections in patients with type 2 diabetes mellitus using machine learning
Source: Front Endocrinol (Lausanne). 2025 Aug 5;16:1593735. doi: 10.3389/fendo.2025.1593735 (PMC12361136; doi:10.3389/fendo.2025.1593735)
Supplement: Supplementary Table 2 — The Validation data of laboratory features. [file Table2.docx]

| **1ASB 2UTIs** | CRP | BACT | UWBCC | ALT | GLU | GGT | Na+ | EOS% | Urea | Cl- | MON | WBC | RDW-CV | AST | URBC | URBCHP | MUCS | UWBCHP | GLO | UNSEHP |
| --- | --- | --- | --- | --- | --- | --- | --- | --- | --- | --- | --- | --- | --- | --- | --- | --- | --- | --- | --- | --- |
| 1 | 1.54 | 1860.90 | 47.20 | 4.60 | 10.24 | 19.20 | 136.50 | 3.10 | 7.20 | 101.50 | 0.73 | 10.44 | 12.40 | 10.90 | 27.30 | 6.83 | 1.50 | 157.10 | 31.90 | 0.13 |
| 1 | 0.47 | 1989.50 | 6.30 | 9.10 | 4.95 | 21.70 | 144.80 | 3.70 | 17.90 | 109.80 | 0.43 | 8.04 | 14.30 | 15.20 | 0.60 | 0.15 | 0.00 | 22.68 | 26.40 | 0.00 |
| 1 | 0.05 |  |  | 14.20 | 5.92 | 14.00 | 143.90 | 1.40 | 6.20 | 109.90 | 0.34 | 4.17 | 12.80 | 16.50 |  |  |  |  | 19.00 |  |
| 2 | 1.67 | 3722.50 | 83.90 | 18.30 | 7.78 | 15.80 | 138.70 | 6.90 | 5.40 | 103.70 | 0.43 | 4.96 | 13.50 | 17.10 | 2.80 | 0.70 | 0.00 | 99.30 | 32.30 | 0.00 |
| 1 |  |  |  | 12.90 | 7.62 | 29.00 | 140.20 | 1.00 | 4.80 | 107.60 | 0.47 | 6.88 | 12.80 | 10.30 |  |  |  |  | 29.70 |  |
| 1 |  |  |  | 8.40 |  | 16.50 | 139.80 | 0.00 | 4.30 | 105.00 | 0.34 | 8.21 | 27.70 | 12.40 |  |  |  |  | 33.50 |  |
| 2 | 0.21 | 2397.30 | 8.80 | 29.20 | 5.51 | 16.30 | 146.50 | 2.40 | 6.00 | 109.10 | 0.25 | 5.89 | 15.20 | 24.50 | 5.80 | 1.45 | 0.00 | 53.73 | 29.90 | 0.25 |
| 1 | 0.03 | 35.30 | 0.50 |  | 8.36 |  | 140.50 | 0.90 | 6.00 | 107.20 | 0.39 | 5.24 | 11.90 |  | 5.20 | 1.30 | 12.70 | 9.63 |  | 0.25 |
| 2 | 2.61 | 17.00 | 1.50 | 13.30 | 9.48 | 27.60 | 132.80 | 0.10 | 9.70 | 97.70 | 1.22 | 13.82 | 13.00 | 18.70 | 1.10 | 0.28 | 0.00 | 7.53 | 24.60 | 0.00 |
| 2 | 0.25 | 835.30 | 35.50 | 27.20 | 13.93 | 23.10 | 138.60 | 2.80 | 9.00 | 104.50 | 0.33 | 5.17 | 12.10 | 23.50 | 6.90 | 1.73 | 1.90 | 125.70 | 26.10 | 0.25 |
| 1 | 0.24 | 2987.50 | 1.50 | 11.60 | 6.34 | 17.30 | 143.80 | 0.10 | 18.20 | 112.60 | 0.38 | 4.56 | 12.40 | 17.50 | 1.70 | 0.42 | 1.00 | 9.13 | 21.30 | 0.38 |
| 1 | 0.15 |  |  | 24.00 | 13.30 | 29.60 | 139.30 | 0.90 | 2.70 | 101.50 | 0.39 | 9.87 | 13.60 | 23.20 |  |  |  |  | 29.90 |  |
| 1 | 0.33 | 8275.70 | 2.30 | 8.10 | 5.94 | 21.40 | 144.50 | 1.90 | 7.60 | 109.50 | 0.49 | 7.59 | 13.00 | 11.10 | 2.80 | 0.70 | 1.20 | 26.88 | 24.30 | 0.30 |
| 1 | 0.29 | 13683.50 | 11.90 | 23.60 | 5.35 | 39.30 | 143.70 | 2.60 | 10.00 | 111.40 | 0.39 | 7.44 | 14.60 | 20.00 | 2.60 | 0.65 | 1.10 | 29.02 | 27.20 | 0.00 |
| 2 | 12.10 | 45.00 | 0.50 | 19.30 | 20.60 | 19.20 | 134.90 | 0.30 | 10.10 | 102.90 | 0.40 | 7.92 | 12.80 | 16.20 | 10.90 | 2.73 | 0.50 | 12.07 | 34.10 | 0.60 |
| 1 | 0.22 |  |  | 14.70 | 5.48 | 14.80 | 142.20 | 0.90 | 4.60 | 110.90 | 0.24 | 4.54 | 12.60 | 19.80 |  |  |  |  | 29.60 |  |
| 2 | 1.79 | 34.00 | 1.90 | 15.30 | 10.89 | 28.10 | 136.70 | 0.10 | 5.60 | 100.60 | 0.41 | 10.59 | 12.90 | 17.30 | 46.00 | 11.50 | 10.20 | 36.95 | 35.10 | 0.38 |
| 1 | 0.70 | 1173.00 | 25.30 | 12.40 | 18.79 | 26.10 | 135.00 | 0.50 | 7.20 | 98.60 | 0.43 | 8.02 | 13.60 | 14.90 | 2.20 | 0.55 | 1.90 | 78.58 | 33.00 | 1.23 |
| 1 | 0.19 | 18764.50 | 0.00 | 9.90 | 5.02 | 12.50 | 142.60 | 1.50 | 4.50 | 104.80 | 0.31 | 8.83 | 13.40 | 17.00 | 0.00 | 0.00 | 2.80 | 2.90 | 22.40 | 0.00 |
| 1 | 0.24 | 2666.00 | 6.30 | 8.80 | 3.26 | 18.10 | 140.90 | 2.40 | 18.30 | 110.00 | 0.33 | 5.02 | 13.40 | 12.90 | 2.30 | 0.57 | 38.00 | 52.73 | 29.50 | 0.00 |
| 1 | 0.30 | 4238.80 | 0.80 | 15.30 | 12.77 | 28.00 | 137.20 | 2.20 | 13.10 | 108.80 | 0.38 | 4.98 | 14.50 | 13.10 | 0.90 | 0.23 | 6.30 | 53.90 | 28.80 | 0.97 |
| 1 | 0.32 | 4181.50 | 5.40 | 13.30 | 8.86 | 28.20 | 140.10 | 1.50 | 6.30 | 105.80 | 0.36 | 8.66 | 12.10 | 14.70 | 1.20 | 0.30 | 7.80 | 57.92 | 28.20 | 0.13 |
| 2 | 8.72 | 480.90 | 0.00 | 25.00 | 6.56 | 216.60 | 134.20 | 0.50 | 11.00 | 105.40 | 0.95 | 13.92 | 14.10 | 23.70 | 6.00 | 1.50 | 3.40 | 0.57 | 36.30 | 0.25 |
| 1 | 14.30 | 7895.30 | 0.50 | 17.20 | 10.09 | 49.90 | 136.10 | 0.10 | 6.40 | 103.50 | 0.58 | 8.32 | 11.50 | 22.90 | 2.80 | 0.70 | 0.00 | 2.77 | 30.30 | 0.13 |
| 1 | 0.15 | 46.30 | 0.00 | 16.70 | 5.91 | 21.40 | 140.40 | 1.10 | 9.00 | 108.80 | 0.25 | 4.54 | 12.30 | 19.70 | 39.70 | 9.93 | 5.80 | 0.70 | 22.80 | 0.00 |
| 2 | 0.20 | 14034.40 | 0.00 | 24.90 | 9.11 | 34.20 | 140.10 | 2.50 | 7.90 | 107.20 | 0.35 | 5.99 | 13.10 | 25.10 | 7.90 | 1.98 | 0.00 | 5.42 | 31.40 | 0.55 |
| 1 | 0.98 | 6477.50 | 0.00 | 10.20 | 9.50 | 30.00 | 144.70 | 5.80 | 7.60 | 109.40 | 0.49 | 6.87 | 13.30 | 16.50 | 4.90 | 1.23 | 1.70 | 2.35 | 23.40 | 0.20 |
| 1 |  |  |  | 10.80 | 12.79 | 20.30 | 134.70 | 1.90 | 11.30 | 103.10 | 0.47 | 5.86 | 12.70 | 9.70 |  |  |  |  | 27.00 |  |
| 1 | 0.07 | 162.50 | 8.80 | 16.60 | 15.17 | 19.90 | 138.90 | 1.70 | 7.90 | 107.80 | 0.31 | 5.13 | 12.60 | 16.30 | 17.20 | 4.30 | 17.50 | 291.75 | 20.00 | 3.27 |
| 2 | 0.71 | 5321.50 | 0.50 | 15.20 | 10.88 | 48.00 | 137.10 | 2.50 | 10.10 | 98.20 | 0.33 | 5.26 | 15.00 | 23.60 | 4.00 | 1.00 | 0.00 | 5.05 | 40.00 | 0.13 |
| 1 | 0.62 | 3809.00 | 1.00 | 34.30 | 6.07 | 68.10 | 142.60 | 3.30 | 6.20 | 105.00 | 0.33 | 5.11 | 13.70 | 19.40 | 10.20 | 2.55 | 8.30 | 28.82 | 24.20 | 0.47 |
| 1 |  | 1634.50 | 10.70 | 23.30 | 16.18 | 52.50 | 134.00 | 0.10 | 25.70 | 101.10 | 0.62 | 15.90 | 12.20 | 11.10 | 4.60 | 1.15 | 0.00 | 47.65 | 30.30 | 0.00 |
| 1 | 0.16 | 9236.30 | 297.90 | 21.70 | 16.10 | 46.00 | 138.20 | 2.70 | 15.60 | 104.10 | 0.37 | 6.74 | 15.20 | 25.10 | 4.70 | 1.18 | 0.00 | 767.20 | 30.80 | 0.25 |
| 2 | 5.38 | 1887.30 | 107.10 | 12.90 | 4.69 | 38.00 | 139.90 | 0.10 | 10.70 | 103.80 | 0.03 | 9.03 | 13.10 | 13.50 | 16.60 | 4.15 | 4.90 | 481.93 | 27.30 | 0.72 |
| 1 | 2.04 | 1744.30 | 1184.20 | 29.60 | 6.96 | 26.60 | 139.10 | 3.20 | 8.80 | 98.10 | 0.67 | 8.31 | 13.20 | 28.30 | 407.90 | 101.97 | 0.00 | 4365.82 | 29.60 | 7.97 |
| 1 | 0.37 | 5592.80 | 19.00 | 9.60 | 14.80 | 14.10 | 136.90 | 1.10 | 17.60 | 106.10 | 0.26 | 5.84 | 11.90 | 14.00 | 1.70 | 0.42 | 1.50 | 58.05 | 27.00 | 0.93 |
| 1 | 0.23 | 4369.50 | 3.90 | 31.90 | 6.68 | 27.50 | 139.60 | 2.10 | 25.30 | 108.90 | 0.36 | 7.17 | 12.80 | 23.80 | 2.30 | 0.57 | 0.00 | 19.93 | 21.20 | 0.33 |
| 1 | 4.11 | 5304.30 | 25.80 | 12.40 | 16.77 | 33.90 | 137.00 | 0.80 | 8.00 | 100.50 | 0.22 | 4.57 | 12.40 | 15.20 | 14.70 | 3.67 | 0.00 | 141.55 | 32.30 | 1.73 |
| 1 |  |  |  | 11.20 | 9.92 | 22.00 | 142.80 | 2.90 | 5.00 | 107.40 | 0.21 | 4.92 | 13.00 | 11.30 |  |  |  |  | 29.90 |  |
| 1 | 0.09 |  |  | 6.40 | 10.81 | 16.80 | 137.10 | 3.70 | 12.80 | 106.70 | 0.29 | 5.30 | 12.50 | 10.10 |  |  |  |  | 20.10 |  |
| 1 | 0.28 | 7.30 | 0.00 | 13.20 | 5.80 | 23.30 | 141.20 | 19.10 | 16.30 | 112.00 | 0.39 | 8.39 | 12.50 | 14.00 | 33.90 | 8.47 | 0.00 | 0.50 | 27.10 | 0.00 |
| 2 | 0.40 | 7684.80 | 4.30 | 12.50 | 11.63 | 27.40 | 140.20 | 1.60 | 5.70 | 105.70 | 0.39 | 7.99 | 13.20 | 15.30 | 1.40 | 0.35 | 0.00 | 44.58 | 30.40 | 0.60 |
| 2 | 0.30 | 1018.50 | 5.40 | 7.30 | 10.06 | 26.60 | 140.30 | 0.90 | 4.90 | 105.20 | 0.27 | 4.90 | 12.20 | 16.90 | 4.40 | 1.10 | 0.50 | 203.35 | 31.30 | 0.85 |
| 1 | 0.27 | 1933.80 | 43.30 | 18.20 | 7.61 | 20.30 | 139.00 | 1.50 | 8.00 | 101.00 | 0.42 | 6.05 | 12.90 | 15.80 | 0.00 | 0.00 | 4.90 | 143.10 | 22.30 | 0.25 |
| 1 | 0.21 |  |  | 15.40 | 4.87 | 13.80 | 141.60 | 0.90 | 7.50 | 106.00 | 0.38 | 8.10 | 13.90 | 15.90 |  |  |  |  | 22.90 |  |
| 2 | 23.70 | 149.40 | 14.60 | 21.20 | 13.30 | 131.40 | 133.60 | 0.20 | 2.60 | 98.20 | 0.57 | 15.96 | 12.40 | 17.80 | 7.10 | 1.77 | 1.90 | 126.72 | 31.10 | 0.00 |
| 1 | 0.22 | 4618.00 | 0.50 | 17.60 | 5.27 | 22.60 | 139.60 | 2.80 | 11.80 | 104.30 | 0.30 | 3.45 | 12.50 | 23.50 | 0.00 | 0.00 | 3.90 | 4.83 | 31.30 | 0.13 |
| 1 | 0.48 | 37.40 | 0.00 | 14.90 | 5.10 | 24.30 | 142.80 | 0.20 | 11.60 | 111.60 | 0.53 | 6.10 | 13.60 | 13.40 | 0.60 | 0.15 | 0.00 | 0.00 | 17.00 | 0.13 |
| 1 | 0.21 | 22.00 | 0.50 | 10.60 | 5.59 | 16.90 | 143.20 | 1.10 | 9.40 | 110.30 | 0.45 | 6.22 | 13.10 | 14.50 | 0.00 | 0.00 | 0.00 | 1.40 | 21.60 | 0.13 |
| 2 | 1.97 | 3149.50 | 0.00 | 13.60 | 6.66 | 31.40 | 136.00 | 4.40 | 8.70 | 101.10 | 0.31 | 6.96 | 12.70 | 16.10 | 0.70 | 0.17 | 0.60 | 3.88 | 29.40 | 0.15 |
| 1 | 0.92 | 1135.50 | 0.00 | 50.10 | 11.45 | 50.50 | 140.10 | 2.60 | 4.30 | 102.80 | 0.39 | 4.86 | 13.10 | 48.40 | 1.10 | 0.28 | 35.50 | 0.13 | 30.40 | 0.00 |
| 1 | 1.63 | 3542.40 | 0.00 | 14.80 | 7.16 | 44.60 | 131.00 | 0.90 | 30.30 | 99.90 | 0.44 | 6.98 | 12.40 | 24.10 | 50.40 | 12.60 | 1.40 | 32.60 | 25.10 | 12.47 |
| 1 | 0.39 | 16757.30 | 0.00 | 16.30 | 6.40 | 11.20 | 140.30 | 8.50 | 7.30 | 105.60 | 0.49 | 11.93 | 12.80 | 14.90 | 0.00 | 0.00 | 0.00 | 11.95 | 22.70 | 0.00 |
| 2 | 1.38 | 2547.70 | 0.00 | 39.60 | 16.20 | 116.60 | 139.40 | 1.00 | 4.50 | 104.20 | 0.50 | 8.18 | 13.10 | 39.40 | 0.60 | 0.15 | 8.80 | 3.85 | 37.10 | 0.25 |
| 1 | 1.33 | 4836.10 | 23.20 | 6.90 | 8.46 | 10.20 | 137.50 | 2.60 | 25.70 | 96.70 | 0.46 | 6.96 | 12.70 | 8.10 | 8.00 | 2.00 | 0.00 | 267.88 | 24.70 | 2.35 |
| 1 |  | 8279.70 | 230.70 |  |  |  |  |  |  |  |  |  |  |  | 23.80 | 5.95 | 0.00 | 3317.70 |  | 0.72 |
| 1 | 0.36 |  |  | 13.30 | 6.85 | 13.60 | 141.40 | 1.50 | 7.50 | 113.30 | 0.14 | 3.38 | 15.40 | 21.20 |  |  |  |  | 31.50 |  |
| 2 | 0.07 | 66.20 | 0.00 |  | 10.39 |  | 139.20 |  | 5.80 | 104.80 |  |  |  |  | 15.60 | 3.90 | 14.10 | 9.75 |  | 0.60 |
| 1 | 0.40 | 2206.30 | 7.80 | 41.70 | 6.23 | 54.20 | 139.00 | 2.70 | 15.60 | 109.20 | 0.41 | 9.04 | 12.50 | 52.70 | 19.10 | 4.78 | 1.00 | 36.00 | 30.50 | 0.13 |
| 1 | 0.30 | 361.70 | 115.80 | 9.50 | 3.83 | 12.30 | 145.10 | 6.50 | 21.20 | 115.80 | 0.34 | 5.43 | 13.10 | 14.90 | 39.40 | 9.85 | 0.00 | 348.13 | 27.90 | 0.72 |
| 1 |  | 3689.50 | 6.80 | 5.90 | 4.20 | 17.90 | 137.70 | 0.60 | 5.20 | 107.00 | 0.23 | 7.59 | 13.90 | 16.70 | 26.10 | 6.53 | 0.00 | 116.33 | 20.30 | 0.85 |
| 1 | 0.43 | 136.30 | 2.40 | 15.40 | 9.89 | 25.70 | 141.40 | 1.20 | 7.60 | 104.20 | 0.33 | 6.80 | 11.90 | 17.50 | 4.40 | 1.10 | 1.50 | 19.88 | 29.40 | 0.13 |
| 2 |  | 37.80 | 0.50 |  | 5.36 |  | 142.40 | 2.20 |  | 103.70 | 0.57 | 6.57 | 14.00 |  | 76.40 | 19.10 | 24.80 | 5.58 |  | 0.13 |
| 1 | 0.60 | 66.70 | 104.70 | 24.40 | 4.84 | 14.70 | 141.30 | 1.10 | 14.50 | 113.90 | 0.21 | 5.70 | 13.90 | 37.70 | 27.80 | 6.95 | 1.50 | 498.05 | 45.80 | 0.97 |
| 1 | 0.42 | 6988.40 | 0.00 | 19.50 | 4.97 | 17.90 | 138.40 | 1.60 | 17.60 | 108.80 | 0.37 | 6.43 | 15.30 | 22.50 | 5.40 | 1.35 | 0.90 | 3.10 | 18.80 | 0.68 |
| 2 | 0.04 |  |  | 13.30 | 6.45 | 8.40 | 146.60 | 0.90 | 4.60 | 111.60 | 0.23 | 4.57 | 13.70 | 20.60 |  |  |  |  | 25.40 |  |
| 1 |  | 3396.80 | 0.70 | 10.30 | 4.37 | 27.40 | 139.20 | 1.30 | 4.20 | 105.10 | 0.35 | 6.43 | 15.00 | 15.90 | 474.00 | 118.50 | 7.50 | 304.02 | 23.60 | 0.38 |
| 2 | 0.20 | 41.30 | 166.00 | 13.30 | 4.48 | 17.70 | 141.90 | 5.20 | 6.10 | 107.20 | 0.35 | 5.66 | 13.00 | 13.90 | 0.60 | 0.15 | 1.00 | 294.10 | 26.40 | 1.23 |
| 1 | 0.48 | 46.30 | 2.40 | 11.90 | 3.98 | 11.20 | 142.60 | 0.90 | 23.20 | 109.60 | 0.47 | 8.14 | 14.60 | 16.50 | 2.90 | 0.72 | 0.50 | 6.85 | 32.40 | 0.00 |
| 1 | 3.03 | 17.00 | 0.00 | 26.70 | 3.94 | 30.50 | 143.40 | 2.40 | 17.50 | 112.00 | 0.61 | 9.12 | 13.40 | 21.70 | 0.60 | 0.15 | 0.00 | 0.00 | 32.60 | 0.00 |
| 1 | 18.70 |  |  | 8.70 | 7.54 | 26.40 | 135.90 | 0.10 | 12.20 | 91.00 | 0.51 | 13.39 | 14.40 | 9.30 |  |  |  |  | 31.00 |  |
| 1 | 0.05 | 4628.50 | 2.40 | 12.50 | 15.05 | 13.60 | 136.80 | 0.30 | 3.50 | 98.40 | 0.20 | 4.76 | 12.90 | 15.00 | 0.70 | 0.17 | 3.70 | 16.75 | 25.50 | 0.45 |
| 1 | 1.08 | 1074.30 | 1.50 | 30.70 | 8.27 | 23.60 | 133.80 | 1.80 | 13.00 | 100.30 | 0.66 | 10.59 | 19.40 | 32.10 | 7.00 | 1.75 | 0.50 | 10.47 | 29.70 | 0.25 |
| 2 |  | 2228.60 | 772.80 | 13.40 | 8.85 | 26.10 | 137.10 | 1.10 | 10.30 | 102.40 | 0.42 | 8.10 | 12.80 | 12.80 | 122.10 | 30.52 | 0.00 | 4352.63 | 27.30 | 11.22 |
| 2 | 0.17 | 20332.40 | 18.60 | 32.80 | 5.70 | 39.60 | 141.00 | 1.40 | 11.70 | 108.70 | 0.36 | 6.37 | 11.80 | 27.30 | 3.10 | 0.78 | 0.00 | 174.47 | 24.50 | 0.68 |
| 1 |  |  |  |  |  |  | 143.20 | 2.60 | 24.10 | 109.60 | 0.36 | 5.13 | 16.80 |  |  |  |  |  |  |  |
| 1 | 0.42 | 7623.80 | 1.10 | 21.90 | 11.33 | 31.30 | 138.70 | 0.80 | 4.60 | 100.80 | 0.38 | 7.90 | 12.90 | 18.10 | 9.20 | 2.30 | 16.80 | 19.82 | 40.10 | 0.55 |
| 1 | 0.13 | 18383.80 | 2.10 | 31.40 |  | 29.00 | 139.20 | 2.00 | 13.60 | 103.80 | 0.30 | 6.46 | 13.20 | 35.40 | 5.00 | 1.25 | 0.00 | 79.85 | 18.70 | 0.53 |
| 1 |  | 266.50 | 0.50 |  |  |  |  | 2.30 |  |  | 0.46 | 5.94 | 11.10 |  | 4.50 | 1.13 | 0.50 | 7.33 |  | 0.25 |
| 2 | 0.18 | 1249.30 | 0.50 | 13.10 | 11.23 | 16.70 | 137.50 | 2.00 | 6.70 | 103.80 | 0.29 | 8.49 | 13.60 | 17.60 | 2.50 | 0.63 | 2.10 | 17.73 | 29.20 | 0.13 |
| 1 | 0.19 | 309.30 | 3.40 | 11.70 | 10.07 | 23.10 | 141.80 | 1.30 | 9.00 | 110.00 | 0.23 | 4.29 | 15.00 | 12.90 | 1.70 | 0.42 | 0.50 | 80.38 | 23.70 | 0.25 |
| 2 | 7.84 | 245.40 | 8.80 | 29.20 | 6.50 | 43.10 | 129.60 | 0.60 | 36.30 | 104.30 | 0.15 | 2.72 | 11.90 | 34.40 | 3.40 | 0.85 | 0.00 | 161.50 | 32.70 | 0.97 |
| 1 | 0.27 | 7052.60 | 2.20 | 15.90 | 5.68 | 16.50 | 144.30 | 0.90 | 3.90 | 110.60 | 0.27 | 3.42 | 14.40 | 23.30 | 3.80 | 0.95 | 0.00 | 13.32 | 22.90 | 0.28 |
| 2 | 1.86 | 4749.70 | 41.50 | 5.70 | 4.17 | 10.80 | 139.70 | 11.60 | 22.10 | 106.80 | 0.15 | 4.36 | 13.80 | 11.10 | 126.80 | 31.70 | 6.10 | 267.90 | 26.60 | 0.78 |
| 2 | 1.59 | 4027.10 | 2.40 | 35.30 | 3.94 | 49.60 | 144.40 | 0.80 | 10.50 | 113.80 | 0.83 | 13.55 | 14.10 | 21.60 | 3.80 | 0.95 | 0.80 | 38.05 | 24.70 | 0.00 |
| 2 | 0.81 | 5927.40 | 132.80 | 9.10 | 13.74 | 27.50 | 141.60 | 3.60 | 5.10 | 103.50 | 0.38 | 7.85 | 13.10 | 17.50 | 6.30 | 1.57 | 0.00 | 827.92 | 30.40 | 1.65 |
| 2 | 3.36 | 2555.00 | 8.90 | 32.60 | 6.52 | 38.90 | 139.30 | 2.80 | 3.60 | 101.40 | 0.32 | 5.26 | 14.60 | 39.10 | 184.10 | 46.02 | 79.30 | 212.25 | 22.10 | 0.13 |
| 1 | 0.50 | 1092.80 | 0.00 | 24.60 | 2.27 | 24.40 | 142.90 | 0.60 | 12.00 | 112.10 | 0.29 | 4.15 | 17.20 | 21.00 | 1.70 | 0.42 | 0.50 | 0.35 | 22.20 | 0.00 |
| 1 | 0.03 | 6369.30 | 1.40 | 23.00 | 6.91 | 56.50 | 140.60 | 0.60 | 7.40 | 113.10 | 0.18 | 3.59 | 29.70 | 16.90 | 4.80 | 1.20 | 5.40 | 39.60 | 22.50 | 0.00 |
| 1 | 0.08 |  |  | 14.50 | 5.82 | 24.20 | 143.50 | 0.80 | 7.10 | 106.80 | 0.36 | 5.73 | 13.50 | 13.80 |  |  |  |  | 26.50 |  |
| 1 |  |  |  |  |  |  | 127.70 | 0.30 |  | 96.90 | 0.52 | 8.21 | 20.00 |  |  |  |  |  |  |  |
| 1 | 0.35 | 15358.50 | 4.40 | 13.90 | 8.68 | 20.60 | 142.00 | 2.80 | 21.20 | 110.90 | 0.53 | 10.53 | 13.50 | 11.90 | 8.50 | 2.13 | 0.00 | 19.43 | 23.60 | 0.00 |
| 2 | 13.40 | 8205.40 | 7.00 | 53.30 |  | 191.10 | 134.60 | 0.30 | 8.90 | 99.20 | 0.65 | 6.03 | 13.70 | 30.40 | 1552.00 | 388.00 | 4.20 | 129.80 | 25.70 | 2.08 |
| 1 | 0.51 | 4793.90 | 0.60 | 7.50 | 3.74 | 16.20 | 143.10 | 4.10 | 26.80 | 103.90 | 0.25 | 3.40 | 15.80 | 11.70 | 1.40 | 0.35 | 1.80 | 9.47 | 28.20 | 0.30 |
| 2 | 7.10 | 580.50 | 21.40 | 9.50 | 12.86 | 12.20 | 137.60 | 0.40 | 16.00 | 99.90 | 0.55 | 9.06 | 11.90 | 14.90 | 1.70 | 0.42 | 1.90 | 41.05 | 34.40 | 0.00 |
| 2 | 2.17 |  |  |  |  |  | 141.40 | 0.50 |  | 103.30 | 0.36 | 6.90 | 15.80 |  |  |  |  |  |  |  |
| 1 | 0.47 | 5366.50 | 10.60 | 9.90 | 3.03 | 21.20 | 144.40 | 2.40 | 22.70 | 116.90 | 0.22 | 5.23 | 12.80 | 11.40 | 3.90 | 0.97 | 2.00 | 55.65 | 18.40 | 0.33 |
| 1 | 0.71 | 1404.30 | 7.30 | 20.90 | 5.39 | 74.40 | 138.80 | 1.80 | 7.00 | 103.90 | 0.39 | 12.90 | 13.30 | 35.70 | 1.10 | 0.28 | 9.20 | 21.18 | 25.60 | 0.13 |
| 1 | 0.05 | 6.00 | 0.00 | 12.30 | 8.94 | 14.40 | 141.10 | 0.80 | 3.60 | 105.80 | 0.27 | 6.50 | 12.10 | 14.80 | 1.10 | 0.28 | 0.50 | 4.10 | 29.20 | 0.00 |
| 2 |  |  |  | 39.90 | 5.52 | 19.20 | 142.00 | 1.20 | 5.70 | 108.30 | 0.30 | 5.97 | 12.80 | 21.20 |  |  |  |  | 27.00 |  |
| 1 | 0.17 | 770.50 | 0.00 | 9.10 | 9.76 | 15.20 | 142.20 | 0.90 | 6.30 | 105.80 | 0.50 | 6.71 | 13.40 | 19.20 | 2.20 | 0.55 | 0.00 | 5.15 | 33.10 | 0.25 |
| 1 | 0.11 | 2408.30 | 2.90 | 16.40 | 13.85 | 10.70 | 138.00 | 3.70 | 6.90 | 105.20 | 0.26 | 5.27 | 12.60 | 17.10 | 0.60 | 0.15 | 0.00 | 14.25 | 31.10 | 0.00 |
| 1 | 0.50 | 4973.60 | 86.00 | 11.50 | 4.50 | 25.60 | 141.90 | 3.50 | 15.30 | 115.40 | 0.28 | 7.04 | 15.20 | 15.40 | 5.60 | 1.40 | 0.00 | 88.80 | 24.60 | 3.95 |
